# Advances in Computer Vision and Sensor-Based Methods for Intelligent Power Transmission Line Inspection

**DOI:** 10.3390/s26154819

**Published:** 2026-07-29

**Authors:** Vasileios N. Kouris, Eleni Vrochidou, George A. Papakostas

**Affiliations:** MLV Research Group, Department of Informatics, Democritus University of Thrace, 65404 Kavala, Greece; vakouri@cs.duth.gr (V.N.K.); evrochid@cs.duth.gr (E.V.)

**Keywords:** computer vision, power transmission line, UAV inspection, defect detection, deep learning

## Abstract

Electricity is fundamental for all modern infrastructures, while disruptions in transmission networks could result in severe failures in healthcare, transportation, communication, industry, and all related to public safety. However, inspection of overhead lines and their components is costly and labor-intensive. The convergence of Unmanned Aerial Vehicles (UAVs), advanced imaging sensors, and deep-learning-based Computer Vision has reshaped this domain. To this end, this work presents a systematic review of Computer Vision applications in electric power transmission line inspection. From an initial 1493 Scopus records, 148 studies published between 2018 and 2026 were retained through a transparent, multi-stage screening and quality-scoring process based on PRISMA guidelines. The reviewed literature was synthesized across four axes: (1) monitoring platforms and sensor technologies, (2) Computer Vision approaches per vision task, (3) datasets, metrics, and evaluation practices, and (4) synthesis of results and industrial adoption. The analysis of the literature confirms the dominance of You Only Look Once (YOLO) family models for real-time edge deployment and the rising adoption of Transformer architectures, while exposing persistent gaps in dataset availability, domain generalization, and field validation. The review concludes with the open challenges and concrete future directions toward fully autonomous, robust, and sustainable inspection systems.

## 1. Introduction

Nowadays, electricity lies beneath all critical infrastructures of modern society, including healthcare, transportation, communication, and industry. Even small interruptions in transmission systems could trigger severe socio-economic failures. Uninterrupted and reliable operation of electric power transmission and distribution networks is, therefore, one of the most important pillars for sustaining and developing the modern economy, industry, and society. As global demand for electricity rises continuously and exponentially, and as the transition to renewable energy sources (RES) accelerates rapidly to address climate change, ensuring stability and integrity of the power grid becomes ever more critical. However, overhead transmission lines and their numerous components, including insulators, metal pylons, high-voltage conductors, vibration dampers, and various fittings, are constantly exposed to a range of extreme weather conditions, continuous mechanical stress, corrosion phenomena, and diverse environmental hazards [1,2]. The aging of this critical infrastructure, combined with growing environmental challenges such as uncontrolled vegetation encroachment and extreme temperatures, can lead to unpredictable failures. Continuous, accurate, and safe inspection of overhead lines is therefore considered essential [3].

Historically, inspection and preventive maintenance of power networks relied exclusively on regular ground patrols by specialized technical crews and, in the case of large-scale networks, on aerial inspections using manned helicopters [3]. Traditional methods, even though effective, are nowadays characterized by significant and challenging limitations; ground patrols are extremely time-consuming, require multiple work hours, and expose employees to danger, especially when networks pass through inaccessible, mountainous, or forested regions. Inspections based on manned helicopters, although faster, remain costly, depend on specialized pilots, and are related to an increased carbon footprint due to fuel consumption and emissions. Moreover, in both cases, visual inspection of components is carried out entirely by human inspectors, making the process directly dependent on the individual’s experience, attention, and subjective judgment [4].

Beyond direct visual observation, several non-visual and hybrid sensing modalities have matured to provide continuous, online monitoring of transmission infrastructure, complementing patrol-based offline inspection [3]. Thermal imaging enables UAVs to identify abnormally heated regions and locate defects from a safe distance, without interrupting the power supply [5]. Airborne and mobile laser scanning further supplies dense, weather- and lighting-resilient three-dimensional measurements that overcome the two-dimensional, line-of-sight constraints of image-based clearance estimation between conductors and encroaching vegetation [6,7]. Electrical and signal-processing techniques based on emitted signals, including operating-condition monitoring of load current, leakage current, and partial discharge [8], as well as wavelet- and Hilbert–Huang-transform-based transient signal analysis [9], analyze live grid signals to detect and localize high-impedance or DC faults instantly, bypassing the latency inherent in post-flight image processing. Additionally, IoT-enabled sensor nodes and smart grid communication infrastructures can support the continuous stream of multiple measurements, enabling real-time monitoring. Together, these modalities address distinct blind spots of both traditional patrol-based inspection and image-based Computer Vision approaches, motivating their combination with visual methods in comprehensive monitoring strategies [3].

Recently, the rapid development of Unmanned Aerial Vehicles (UAVs), namely drones, combined with the corresponding evolution of digital imaging sensors, such as visible-spectrum red/green/blue (RGB) cameras, thermal/infrared cameras, and Light Detection and Ranging (LiDAR) laser sensors, and the maturation of Computer Vision and Artificial Intelligence (AI) algorithms, has revolutionized the field of transmission line inspection. UAV platforms constitute flexible, low-cost, and safe solutions for collecting high-resolution multi-modal data without endangering personnel’s safety [10]. At the same time, the application of deep learning algorithms, especially Convolutional Neural Networks (CNNs) [11], as well as the more recent Transformer-based architectures, such as DEtection TRansformer (DETR) [12], enable automatic detection and classification of components, allowing for defect recognition, e.g., broken insulator discs, extensive corrosion of metal parts, broken conductor strands, etc.

Despite the rapid progress of scientific research in this specific area, broad industrial adoption of fully autonomous Computer Vision systems still faces certain critical obstacles. Contemporary challenges include the extremely high complexity of visual backgrounds (cluttered backgrounds), where network components are visually confused with the natural landscape, rapidly changing weather and lighting conditions, dramatic variation in size of objects under examination (multi-scale targets) ranging from gigantic pylons to tiny bolts [13], and the strict computational constraints of edge-computing systems embedded on the UAVs themselves [14]. Additionally, the ongoing lack of large, class-balanced, high-quality public datasets significantly obstructs the training of robust models capable of generalized performance in different geographical environments (domain generalization) [15].

To this end, the primary objective of this work is to conduct an in-depth, critical, and rigorously systematic literature review of the applications of Computer Vision (CV) in the inspection of electric power lines. Preferred Reporting Items for Systematic Reviews and Meta-Analyses (PRISMA) methodology is employed, resulting in a total of 148 scientific works published in peer-reviewed international scientific journals and conferences during the period 2018 to 2026. This exhaustive review focuses strategically on mapping the following:

- The various monitoring platforms (e.g., autonomous drones, flying robots, fixed cameras, helicopter-mounted sensors) and sensor technologies (RGB, infrared (IR), LiDAR, multispectral) used to collect visual data.
- The advanced Computer Vision approaches employed for a wide range of tasks, spanning from micro-defect detection on components and the estimation of safety distances via three-dimensional methods to thermal analysis and infrared fault diagnosis.
- The available datasets (open and private), the established evaluation metrics, and the industrial Key Performance Indicators (KPIs) that define the success of a method.
- The practical challenges that arise when deploying these algorithms in real time, in resource-constrained environments, as well as the next steps required for their universal adoption by industry.

The ambition of this work is to serve as a complete, comprehensive, and well-documented guide for both academic researchers and engineers in the energy sector, highlighting the state-of-the-art, revealing existing research gaps, and proposing specific directions and solutions for the smooth transition toward the creation of fully autonomous, intelligent, and infallible power network inspection systems. The rest of the paper is structured as follows. Section 2 identifies research gaps in the literature and highlights the contributions of this work. Section 3 presents the proposed research methodology, while results are presented in Section 4. Discussions and conclusions are provided in Section 5 and Section 6, respectively.

## 2. Related Works and Contribution

Although several reviews have already been published in the broader field of power-network inspection [16,17,18,19,20], their careful study reveals specific, systemic limitations that the present review explicitly seeks to address. Specifically:

- Lack of Systematicity and Critical Assessment: Most of the previous related review documents are limited to a descriptive listing of published algorithms and architectures. They do not follow rigorous, recognized protocols (such as PRISMA) for collecting the literature, introducing selection bias. Moreover, they are non-reproducible and fail to comparatively quantify the performance of various models.
- Narrow, Limited Focus: Most of the previous related works concentrate on the detection of a single component (typically insulators) or completely ignore alternative sensor technologies, mainly analyzing optical (RGB) data. Thus, they overlook the strong, ever-increasing trend toward “Data Fusion” from thermal, multispectral, and ultraviolet (UV) cameras, as well as LiDAR data, which often function complementarily.
- Disregard for Real-World Deployment Conditions (Industrial Deployment Gap): A critical gap in previous related works is the absence of assessment of the practical, industrial applicability of the examined models. They evaluate theoretical architectures without examining whether these models have been deployed and validated in the field. They completely overlook technical parameters of vital importance to industry, such as the processing rate on small-form-factor computers, energy consumption, and the model’s total parameter count.
- Ignoring Generalization and Data-Quality Issues: A gap has been identified in the analysis of the robustness of the examined models. It is rarely examined how networks behave under adverse weather conditions (studies in fog, rain, snow) or whether they can generalize their predictions to transmission networks of different countries (domain shift). Furthermore, few reviews highlight the importance of innovative solutions for the lack of fault data, such as Synthetic Data Generation via Generative Adversarial Networks (GANs) and Few-Shot Learning.

Based on the examination of previous related review works, the present review has been designed to fully address the identified gaps. It aims to offer a holistic perspective, critically analyzing not only the theoretical capabilities of the algorithms but also linking them inextricably to hardware technology (sensors, UAVs, edge boards), the available data, and the real, relentless field requirements for the safety and efficiency of the electric power industry.

## 3. Research Methodology

Both the scientific validity and reliability of the conclusions of a literature review depend directly on the transparency, rigor, and reproducibility of the research methodology followed during searching, selecting, and assessing the identified works. Within this framework, the present review was conducted following the guidelines of PRISMA 2020 [21]. The PRISMA framework was chosen for its excellent suitability in synthesizing heterogeneous evidence across interdisciplinary fields, and in particular in domains that combine computer science (Computer Vision, deep learning), electrical engineering, and large-infrastructure management.

The overall review process was organized strictly into the standardized PRISMA phases: Identification, Screening, and Inclusion, with detailed and explicit documentation of all decisions at each individual stage, aiming to minimize the risk of selection bias, allowing full traceability of excluded studies, and reinforcing methodological rigor as expected from a systematic review of this scope.

### 3.1. Identification: Search Strategy and Databases

Literature research was conducted in the Scopus database, selected for its extensive indexing of peer-reviewed scientific journals and conference proceedings in the fields of engineering and computer science [22]. All searches were executed on 7 December 2025. The search process was carried out as a single, fixed run, and no further updates or refreshes were performed afterwards.

To ensure systematic coverage of the field, multiple thematically structured search queries were used, aiming to target inspection platforms, sensor technologies, Computer Vision methodologies, datasets, evaluation metrics, and defect-detection performance. Specifically, the queries (Q) that were executed are included in Table 1.

### 3.2. Screening: Eligibility

All queries were restricted as follows:

- Publication years: 2018–2026;
- Publication stage: Final;
- Subject areas: Engineering (ENGI) and Computer Science (COMP);
- Document types: Articles and reviews;
- Language: English;
- Source type: Journals.

Moreover, studies were included if they cumulatively met the following conditions:

- They focused on overhead power transmission or distribution infrastructure, including lines, pylons, poles, insulators, conductors, fittings, or vegetation near the lines.
- They used optical sensors, such as RGB, thermal/infrared, multispectral, hyperspectral, LiDAR, or 3D point clouds.
- They addressed a Computer Vision task, such as component detection, defect detection, vegetation-encroachment monitoring, safety-distance/sag estimation, or the detection of thermal/ultraviolet anomalies.
- They provided empirical evaluation with quantitative or clearly defined qualitative results.

At this point, the Scopus search returned a total of 1493 records. All records were imported into the Zotero 9.0.5 reference-management software, which was used to remove duplicates (deduplication) based on identical titles, since different queries returned the same documents. This process removed 693 duplicate records, leaving 800 unique studies.

A total of 800 records underwent title screening. Of these, 150 studies were excluded due to obvious irrelevance to the subject, leaving 650 studies for abstract screening. Abstract screening excluded an additional 291 studies for the following reasons:

- Not relevant to transmission line inspection.
- Not based on Computer Vision or non-optical.
- Insufficient experimental detail.
- Issues with publication, type, language, or year.

This stage resulted in 359 eligible studies. Of the 359 eligible studies, 9 were excluded due to the unavailability of the full text (open access). The remaining 350 studies underwent full-text assessment.

**Figure 1:**
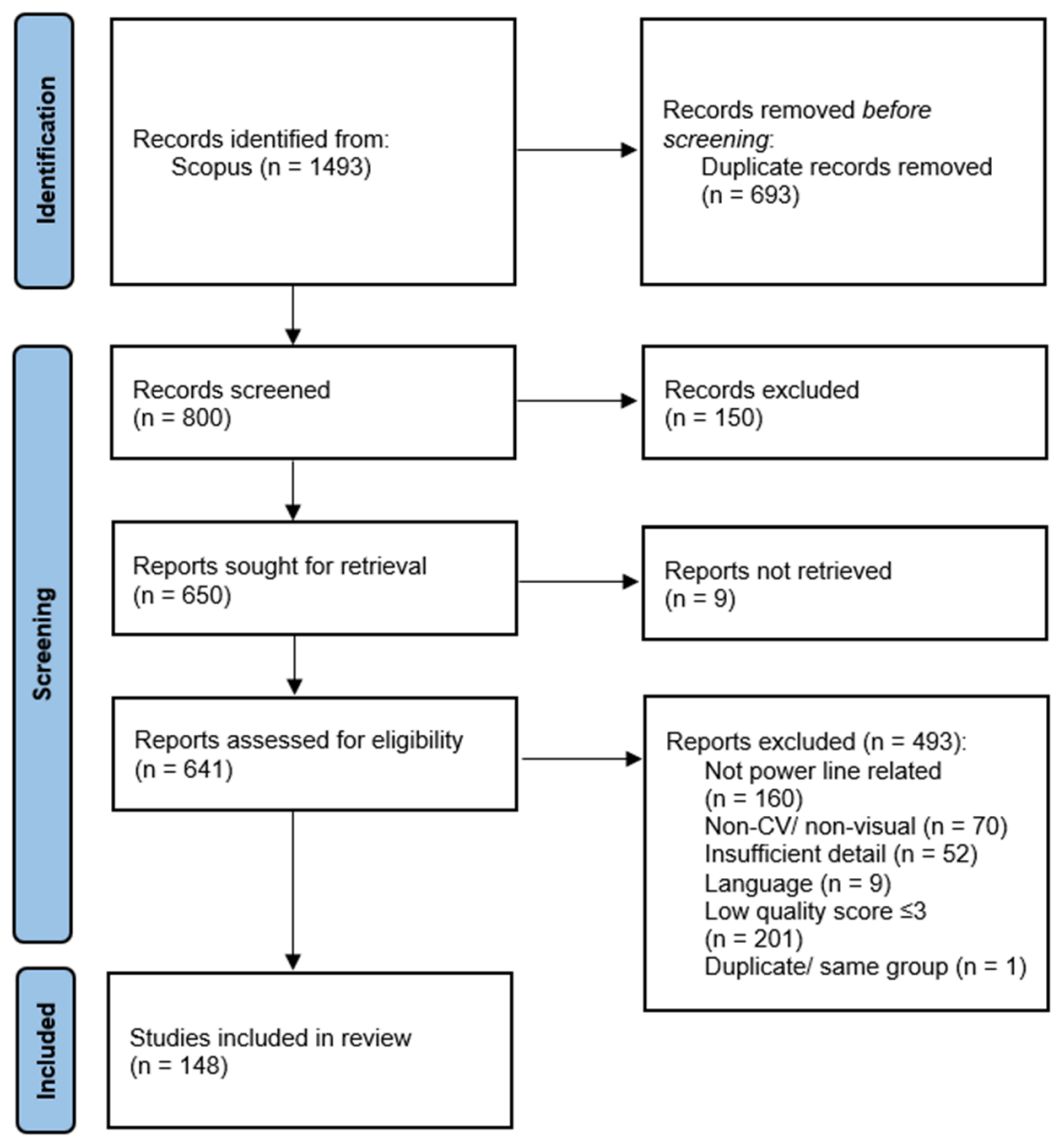
PRISMA 2020 flow diagram of the study identification, screening, eligibility, and inclusion process.

A structured five-point contribution scoring system was applied, which assessed dataset quality, methodological rigor, and validation practices. Only studies with a score of 4 or 5 were retained. As a result, 201 studies were excluded. During the final consolidation of references, one further duplicate article originating from the same research group was identified and removed, resulting in a final corpus of 148 studies. The complete process is illustrated in Figure 1.

### 3.3. Inclusion, Metadata, and Data Synthesis

A structured data-extraction protocol was applied to all 148 included studies. Specifically, the following items were extracted:

- Bibliographic metadata (authors, year, publication type);
- Study design and inspection platform (UAV, ground-based, satellite, or multi-platform);
- Computer Vision methodologies applied;
- Sensors used;
- Dataset characteristics (public, proprietary, synthetic);
- Reported performance metrics;
- Key findings and contributions.

A narrative synthesis was adopted in order to accommodate the methodological heterogeneity observed across the studies. A comparative analysis was carried out to identify performance trends across different datasets and inspection platforms, as well as gaps in current research.

The quality assessment followed a structured, checklist-based approach, consistent with the principles of PRISMA. Each study was assessed according to the following:

- Methodological rigor;
- Openness and reproducibility of the dataset;
- Real-world applicability and industrial validation.

Quantitative performance metrics (e.g., precision, recall, F1-score, etc.) and task-specific measurements (e.g., distance estimation error, etc.) were incorporated into the assessment to support objective comparison. Although a narrative synthesis was used to present the findings, the underlying assessment of the quality of evidence was fully systematic and transparent.

## 4. Synthesis of the Results

Details of the selected literature covering aspects such as advantages, disadvantages, deployment suitability, typical accuracy, complexity, and computational time are provided in Table S1, as Supplementary Materials, driving discussions and comparisons between the reported methods.

Based on the historical context and literature provided across the research works, the technology for electrical transmission line inspection has undergone a significant multi-stage evolution. This evolution transitions from high-risk manual labor to classical Computer Vision, and ultimately to edge-deployed, multi-modal deep learning architectures.

**Figure 2:**
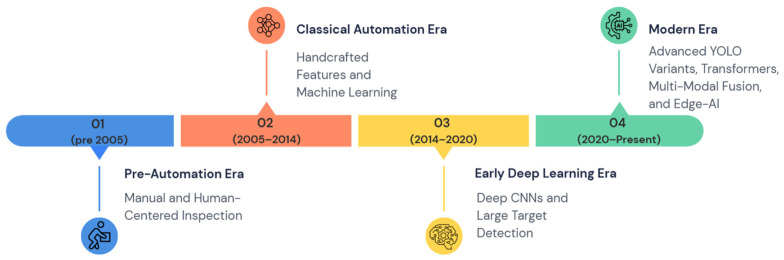
Evolution of power line surveillance.

The evolution of power line surveillance can be organized into four distinct eras, illustrated in Figure 2.

Before the integration of Computer Vision, utility companies relied exclusively on manual monitoring protocols to maintain grid stability. Foot patrols equipped with binoculars, helicopter-assisted manual surveys, and inspection specialists physically climbing live high-voltage towers secured by ropes were among the most common methods. This era was heavily restricted by extreme safety hazards (risks of electrocution or falls), massive economic costs, slow operational speed, and low detection rates caused by human operator fatigue and subjectivity [23,24].

With the introduction of digital cameras and early UAVs, researchers sought to replace manual analysis with automated image processing. Feature extraction relied entirely on manual engineering. Algorithms utilized mathematical and geometric patterns such as edge detection, morphological segmentation, thresholding indices, the Hough Transform, and the Radon Transform to map out power lines and fittings [25,26]. Classifiers like Support Vector Machines (SVMs) or template matching were frequently used to categorize fault status [27]. However, these systems possessed very weak generalization capabilities. They were highly sensitive to environmental noise, changing lighting conditions, and background clutter, which led to high false alarm rates in complex terrains [28].

The breakthrough of deep CNNs shifted the paradigm from handcrafted features to automated end-to-end feature learning. Two-stage object detectors such as Faster R-CNN and early single-stage architectures like Single Shot MultiBox Detector (SSD), YOLOv3, and YOLOv4 became mainstream for isolating key infrastructure components [29,30]. Research focused heavily on locating and extracting larger assets, such as full tower structures and clear insulator strings. Specialized modules like Feature Pyramid Networks (FPN) and basic attention mechanisms began appearing to address multi-scale variations in aerial imagery [31].

Current state-of-the-art frameworks have evolved to solve highly granular defects, e.g., cotter pin missing, strand breakages, or corona ring tilts, under severe adverse conditions. Object detection has expanded from standard CNNs into real-time end-to-end Transformers, such as DETR, RT-DETR, and highly optimized lightweight architectures (YOLOv7, YOLOv8, YOLOv9, YOLOv11) featuring advanced coordination attention mechanisms like SimAM or Bi-level routing attention [32,33]. Modern inspection strategies transcend RGB images by fusing optical streams with Infrared/Thermal imaging to detect abnormal thermal defects, alongside Airborne/Mobile LiDAR point cloud segmentation to monitor vegetation encroachment corridors [5,34]. To mitigate the extreme class imbalance of real-world fault data, modern pipelines implement sophisticated weather-domain transfer generative models to synthesize high-fidelity images of defects shrouded in extreme fog, rain, or snow [35,36]. Instead of collecting data for offline ground-station post-processing, modern workflows optimize neural networks via network pruning, structural distillation, and Reinforcement Learning (RL) to enable on-board, real-time edge processing directly on UAV-embedded computing platforms like NVIDIA Jetson boards [37,38].

**Figure 3:**
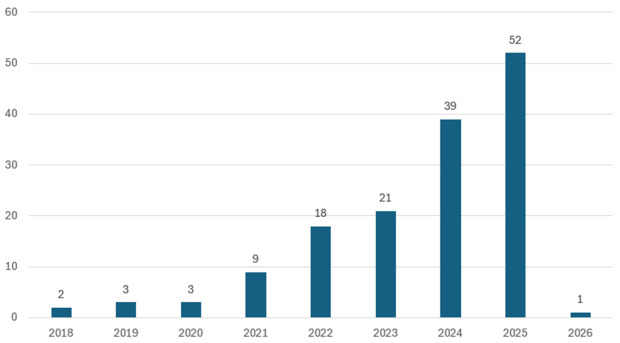
Distribution of identified literature per year.

Additionally, temporal analysis of the 148 reviewed works, illustrated in Figure 3, shows a rising number of papers per year, which directly aligns with the technological advancements of the last two identified eras, highlighting that innovations in sensing, modeling, and deployment guided the ever-increasing research interest. Note that while the search window was between 2018 and 2026 (research executed on 7 December 2025), one early-access article formally dated 2026 was also captured.

**Figure 4:**
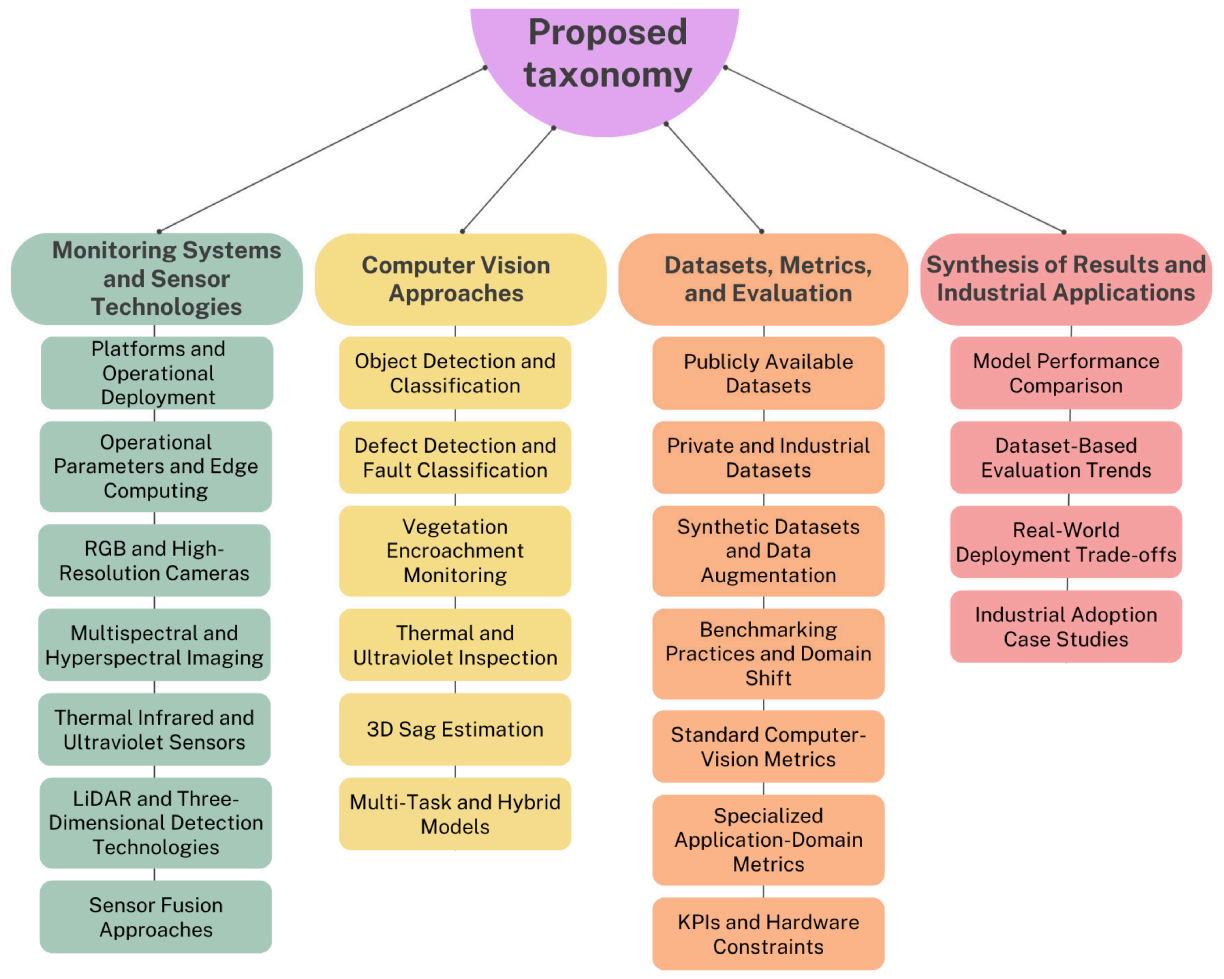
Proposed taxonomy.

In the following, the findings of the 148 reviewed studies are presented across four identified thematic axes, illustrated in Figure 4: monitoring systems and sensor technologies that supply the visual data, Computer Vision approaches applied per vision task, datasets, metrics and evaluation practices that underpin the reported performance, and the overall synthesis of results together with the current state of industrial adoption.

These four broader clusters are formulated based on the keywords of the selected reviewed studies, which span four distinct functional domains. Importantly, these clusters are both conceptually and quantitatively grounded; keyword extraction from 148 articles yields several hundred unique terms (478 different terms), which naturally aggregated into four dominant groups based on frequency and co-occurrence patterns across the selected literature.

Specifically, the “Monitoring systems and sensor technologies” cluster focuses on how data is acquired, based on keywords that describe hardware, sensing modalities, and operational conditions, which is the first stage of all inspection pipelines. The “Computer vision approaches” cluster focuses on algorithms, architectures, and learning paradigms, indicating how the data is being processed, which is the core algorithmic stage of the pipeline. The “Datasets, metrics and evaluation” cluster refers to data resources, annotation challenges, and evaluation protocols, describing how algorithms are validated, which is the evaluation stage of the research pipeline. Finally, the “Synthesis of results and industrial applications” cluster refers to real-world defect types, operational workflows, and industrial deployment, focusing on the deployment and impact stage of the pipeline.

Therefore, the four selected taxonomy clusters aim to reflect the entire lifecycle of inspection research as evidenced directly by the keywords used across the papers, spanning data acquisition, data interpretation, performance validation, and operational deployment. The selected taxonomy aims to indicate the natural structure of the field and to provide a clean, hierarchical organization, appropriate for a systematic review.

### 4.1. Monitoring Systems and Sensor Technologies

The selection of the appropriate monitoring platform and the corresponding optical sensor is not merely a technical detail but the fundamental factor that determines both the quality of training data and the final operational capability and effectiveness of Computer Vision systems in the field [39]. Hardware technologies that shape modern inspection systems, as reflected in the 148 studies of this review, are analyzed in the rest of the section.

#### 4.1.1. Platforms and Operational Deployment

**Figure 5:**
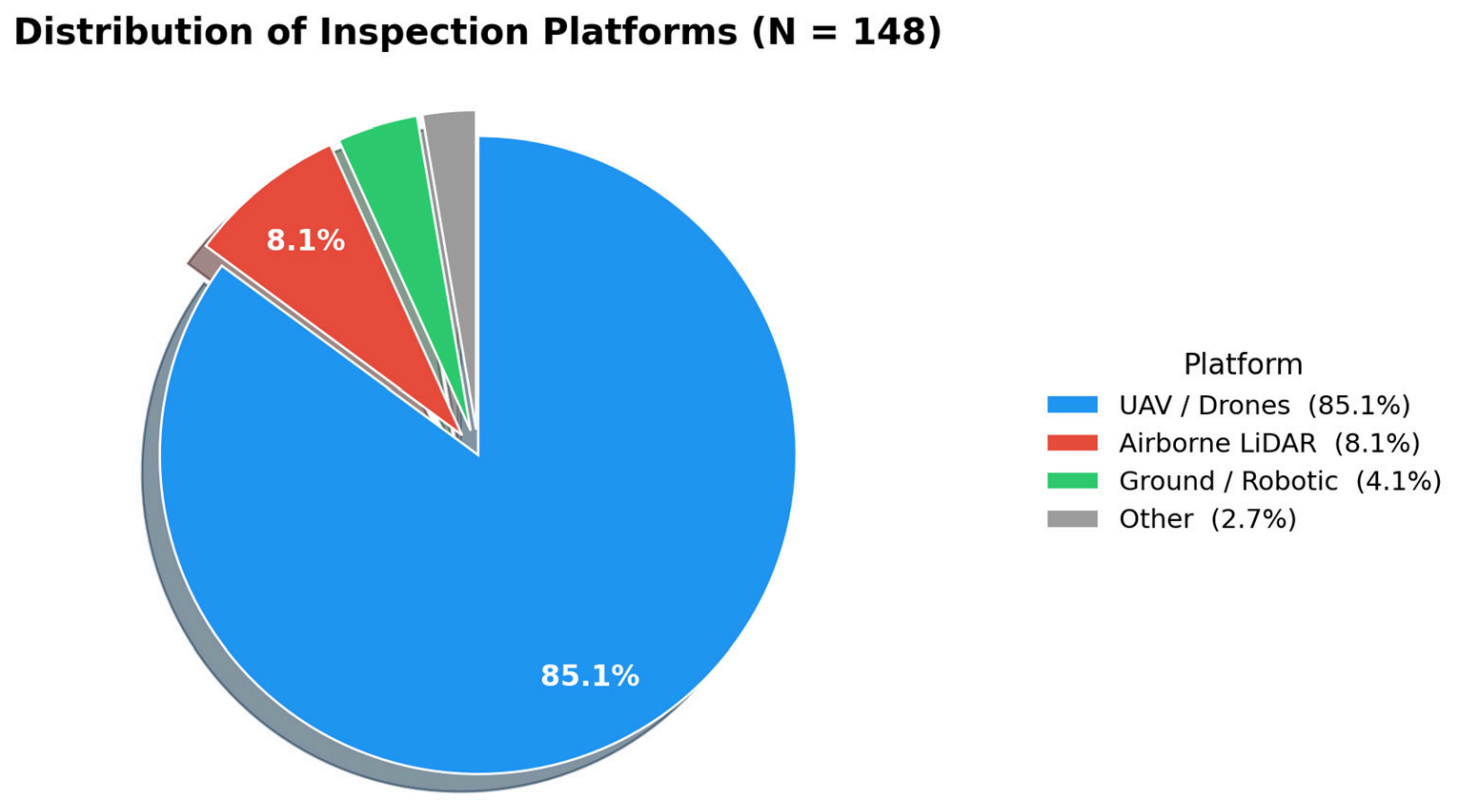
Percentage distribution of inspection platforms across examined literature, highlighting the dominance of UAVs over alternative aerial and ground-based solutions.

Based on the aggregate findings of the literature, UAVs are undeniably the dominant inspection platform across the examined literature, representing over 85% of experimental setups, as seen in Figure 5. The massive preference for UAVs is due to their unparalleled freedom of movement, their hovering capability, and their ability to approach extremely inaccessible components, such as the tops of pylons, from multiple viewing angles without exposing human personnel to danger [9,40,41,42,43,44,45,46].

Alongside human-piloted drones, recent years have seen a rapidly growing trend toward the use of fully Autonomous Aerial Vehicles (AAVs). These vehicles are capable of executing predefined, large-scale flight routes (Beyond Visual Line of Sight, BVLOS), relying on real-time line-detection algorithms (visual servoing/line tracking) that keep the drone centered over the conductors without human intervention [47,48].

Beyond classic aerial platforms, the conducted research also highlights certain specialized robotic configurations. A characteristic example is the “flying–walking” robots, which are designed to fly up to high-voltage lines, “land” smoothly on the conductors, and then roll or walk along them [49]. Such hybrid robots ensure extremely stable shots from a distance of a few centimeters, eliminating the problem of motion blur that often afflicts in-flight drones. Finally, for monitoring low-voltage urban networks or for detecting emergency hazards around the network, e.g., falling trees, illegal construction, or fires, fixed surveillance cameras placed at strategic points, namely surveillance nodes, are often used, as well as systems mounted on the roofs of ground patrol vehicles [50].

#### 4.1.2. Operational Parameters and Edge Computing

Operational deployment of autonomous vision systems on UAVs stumbles over a fundamental, practical obstacle: the severely limited battery autonomy and dramatically low computational power of devices. Transmission of ultra-high-resolution (4K) live video to a remote data center (cloud) for processing is often impossible due to the lack of broadband (5G) networks in the remote, mountainous areas through which transmission lines pass. Consequently, image processing must necessarily be carried out “on-board”.

For this purpose, edge-computing boards are used, with the NVIDIA Jetson series, specifically the Nano, TX2, Xavier NX, and the most recent Orin models, monopolizing the literature, followed by Raspberry Pi 4 microcomputers. Due to the low memory (usually 4 GB to 8 GB RAM) and the strict thermal constraints of these devices, researchers are forced to apply aggressive neural network (NN) compression techniques. Techniques such as network pruning, quantization (from 32-bit float to 8-bit integer), and knowledge distillation, in which a “heavy” teacher model trains a “light” student model, are necessary in order for the system to achieve the critical recognition speed of over 30 frames per second (FPS), ensuring real-time obstacle avoidance [23,51,52,53,54,55,56,57,58,59].

#### 4.1.3. RGB and High-Resolution Cameras

**Figure 6:**
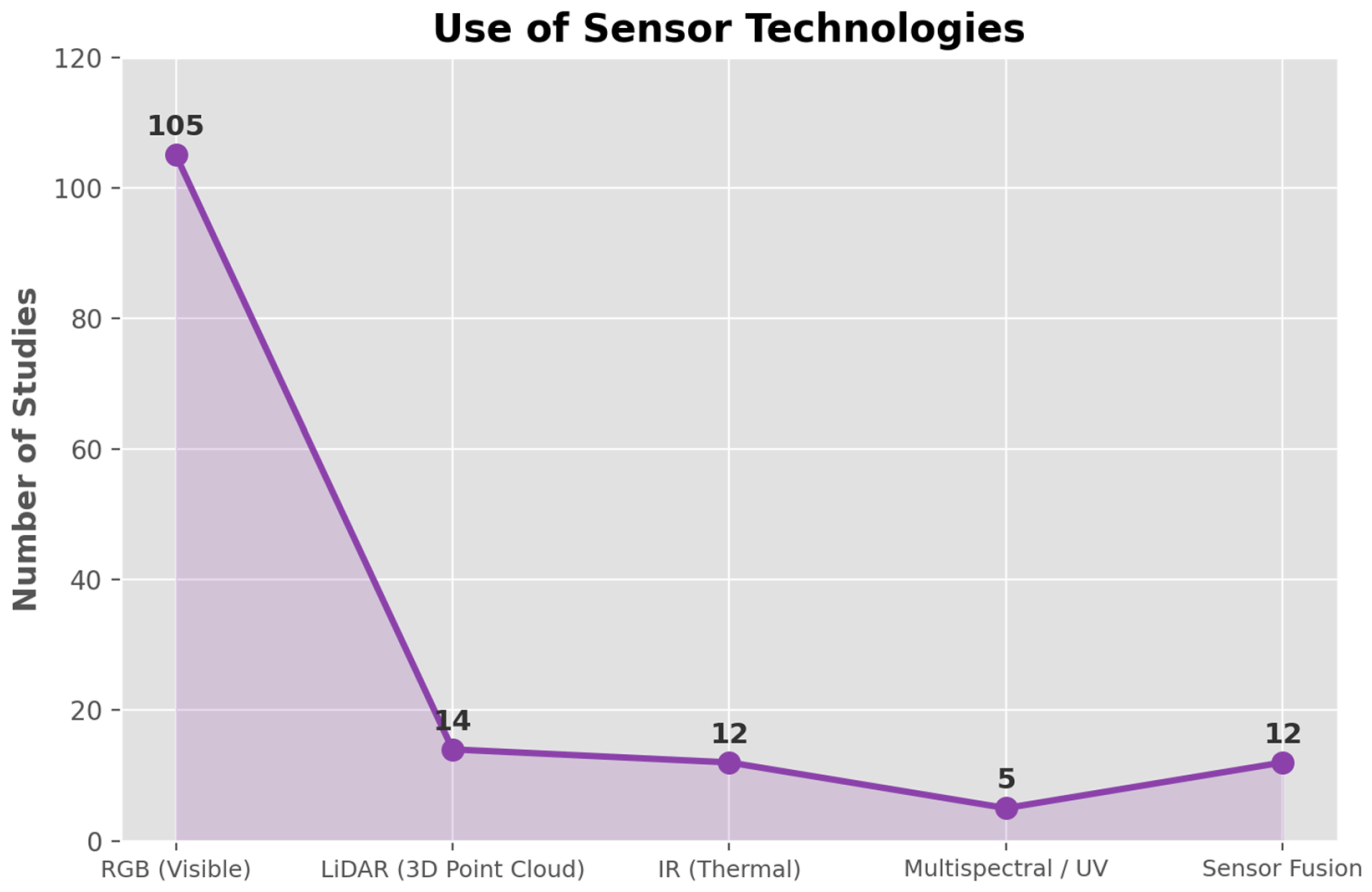
Frequency of use of sensor configurations, indicating the industry’s broad reliance on visible-spectrum (RGB) cameras and the gradual, emerging adoption of multimodal systems (Sensor Fusion) and LiDAR for increased robustness under extreme conditions.

Figure 6 summarizes the use of sensor technologies as identified by the examined literature. As shown in Figure 6, visible-spectrum (RGB) cameras are by far the most widespread sensors in power infrastructure inspection. Their dominance is easily explained: they offer extremely low acquisition costs, are lightweight, integrate seamlessly into any type of drone, and, most importantly, provide incomparably rich visual information on color and fine texture. Thus, they are widely and successfully used for detecting defects in porcelain and glass insulators, such as cracks, breakage, or complete disc loss, for diagnosing wear (rust) on vibration dampers, and for detecting foreign, potentially dangerous objects, e.g., bird nests on pylons, tangled kites, or plastic bags [60,61].

However, despite their usefulness, RGB sensors suffer from a critical, inherent disadvantage: their absolute dependence on external lighting. Their performance and, consequently, the accuracy of the algorithms they feed decreases dramatically under low-light conditions, i.e., during night flights, in the presence of extreme reflections (glare) from the sun on metal cables, or during intense weather phenomena that cause optical scattering, such as dense fog, heavy rain, and snowfall [62].

#### 4.1.4. Multispectral and Hyperspectral Imaging

Although statistically less common in the examined literature, the use of multispectral and especially hyperspectral imaging is gradually beginning to gain ground in specialized industrial applications. These sensors record data in dozens or even hundreds of narrow spectral bands, beyond visible light, allowing the system to recognize material “signatures” that are not at all visible to the naked eye. For example, hyperspectral imaging allows the perfectly accurate spectral separation of the background, such as soil and vegetation types, from metal components, even if they are exactly the same color to the human eye. In addition, it is used in a pioneering way to assess invisible chemical corrosion in aluminum cables at a very early stage [63].

#### 4.1.5. Thermal Infrared and Ultraviolet Sensors

Optical fault detection is sufficient only when defects are structural and have reached the surface. To prevent faults before they even become visible, thermal (IR) cameras are absolutely irreplaceable. They are used to diagnose critical faults that cause local overheating, namely hotspots, due to increased electrical resistance, as occurs in loose cable connections or during internal electrical discharges within composite (polymer) insulators [5,26,64,65,66]. The main problem with thermal data is that it has inherently much lower resolution than the corresponding RGB images, is often blurry (lacking crisp edges), and has a reduced signal-to-noise ratio (SNR), which requires specially adapted neural networks to detect small “hot” targets.

On the other hand, ultraviolet (UV) cameras, although rare in the deep learning literature, mainly due to their extremely high acquisition cost, are used for the visual detection of corona-discharge phenomena, that is, the microscopic discharge of current into the air, which emits ultraviolet radiation and is an infallible indicator of the imminent failure of an insulator or conductor.

#### 4.1.6. LiDAR and Three-Dimensional Detection Technologies

Laser scanners, whether used on the ground in cars (Mobile Laser Scanners) or mounted on UAVs (Airborne Laser Scanners), emit millions of laser pulses per second and produce extremely dense and accurate “point clouds” of physical space. Their main and undisputed use in the power sector lies in the precise, three-dimensional mapping of the entire network corridor. LiDAR makes it possible to estimate conductor sag under real conditions, as well as perhaps the most critical safety application: detecting the safety distance from surrounding vegetation (vegetation-encroachment risk), with centimeter-level accuracy [6,34,67,68,69,70,71].

The greatest challenge in using LiDAR data with NN is dealing with the enormous, often class-imbalanced volume of data. In a typical point cloud of a corridor, the points corresponding to the thin cables occupy less than 1–2% of the total, while the remaining 98% belong to the background, i.e., ground and trees.

#### 4.1.7. Sensor Fusion Approaches

To overcome the individual limitations of each sensor, the cutting edge of research has turned toward Data Fusion, i.e., Sensor Fusion, techniques. The most popular and practical application is the direct combination of RGB and IR data. In this approach, the neural architecture, e.g., AFRNet [72], extracts the rich spatial and color detail from the optical camera and fuses it in real time with the thermal signature from the infrared camera. This multimodal system allows the drone to operate flawlessly both during a sunny day and in the dark of night or under dense fog.

In addition, within the examined literature, there are extremely innovative and hybrid fusion methods, such as the simultaneous use of optical cameras with Digital Radiography (DR) systems. This combination allows the inspection robot not only to see the external surface of the fitting but also to precisely detect the invisible, internal cuts in the core of aluminum-conductor composite-core (ACCC) cables [73,74].

Despite these advantages, the practical implementation of sensor fusion introduces several engineering challenges that are rarely discussed in detail. A recurring issue is resolution mismatch between the fused modalities: infrared sensors typically capture at substantially lower resolution than their optical counterparts (e.g., 640 × 512 IR versus 1920 × 1080 RGB, or 576 × 325 IR versus full-HD RGB), which complicates pixel-level alignment and forces a compromise, most commonly resizing both modalities down to a small, uniform resolution such as 256 × 256 or 128 × 128 pixels for standardized processing [64,65]. This downscaling is particularly problematic for small components such as insulators, since spatial detail that could distinguish a genuine defect from background clutter is discarded before fusion even occurs. Registration itself is non-trivial; one study [47] addressing binocular RGB fusion for thin power line conductors reports that finding matching points across camera pairs is complicated by the multi-pixel width of the line itself and by parallax-induced discrepancies between the two visual fields, requiring skeleton extraction via non-maximum suppression and a recalculated, slope-based matching range to establish a reliable common field of view. A related stereo-vision study reports that noise-filtering steps applied during matching can inadvertently remove valid data points along thin conductors, leaving the reconstructed point cloud insufficiently dense for reliable downstream processing [17].

Beyond alignment, the computational burden of fusion is repeatedly cited as a barrier to onboard, real-time deployment. Combining hyperspectral or LiDAR data with optical imagery increases both payload weight and data volume beyond what airborne embedded processors can handle in real time, a limitation that most reviewed studies acknowledge but leave unresolved, typically by defaulting back to single-modality RGB processing [3,17,75]. Proposed solutions are mainly architectural rather than algorithmic, such as offloading depth or LiDAR processing to ground-based or cloud infrastructure rather than resolving it onboard [17]. Taken together, these findings indicate that sensor fusion in this domain remains constrained less by the extraction of complementary information from multiple modalities and more by the practical mismatch in resolution, geometry, and computational demand between the sensors themselves, precisely the class of implementation-level problem that the high-level framing above does not capture.

### 4.2. Computer Vision Approaches

**Figure 7:**
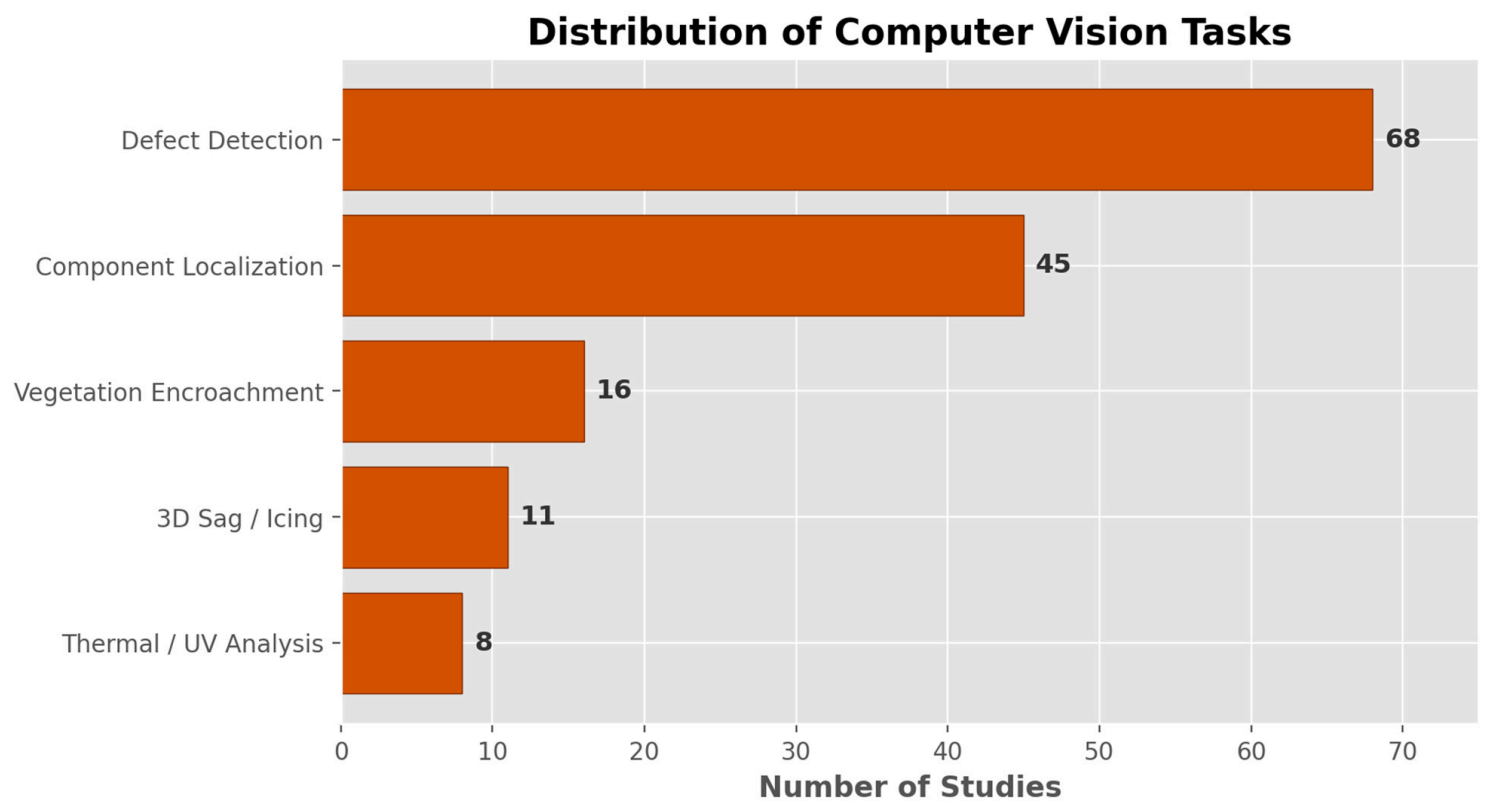
Classification of the examined literature according to the main vision task. A strong concentration is observed on component-level defect detection, as opposed to macroscopic vegetation monitoring and 3D distance measurements.

The spectacular development and evolution of Computer Vision algorithms, and in particular deep learning methods, have enabled the transition from simple, passive digital recording and storing videos and photographs, to the intelligent, “active”, and automated analysis of infrastructure in real-time. This section aims to analyze the main approaches recorded in the literature, classified according to the main vision task they are employed to perform. Figure 7 illustrates the distribution of the reviewed studies across these vision tasks, while Table 2 summarizes, for each task, the prevailing sensors, the dominant methods and architectures, the principal evaluation metrics, and representative studies.

#### 4.2.1. Object Detection and Classification

The most fundamental and frequently encountered task in modern inspection systems is the automatic localization and classification of the numerous power network components, such as insulators, vibration dampers, corona rings, metal pylons, and fittings, within extremely high-resolution aerial photographs, which are characterized by complex natural backgrounds [96,97].

The methods proposed in the literature rely almost exclusively on modern Object Detection architectures. Particularly popular in the literature are the “one-stage detectors”. The You Only Look Once (YOLO) family of algorithms, starting from classic applications of YOLOv3/v4 [27] and extending to the most advanced versions YOLOv7, YOLOv8, and YOLOv11 [60,76], dominates absolutely due to its unique ability to operate seamlessly in real time [77,78,98,99,100,101,102,103,104,105].

In contrast, “two-stage detectors”, such as the classic Faster R-CNN architecture, although historically offering marginally higher precision in detecting borderline details [81], are gradually being abandoned or restricted to offline analyses at control centers, since they lag dramatically in speed, making them unsuitable for deployment directly on drones.

To successfully address the complexity of the natural background where green trees are confused with glass insulators, and the extremely variable scales of objects within the same image, researchers have proceeded to extensive, specialized modifications of base models. Incorporation of attention mechanisms such as Convolutional Block Attention Module (CBAM), Coordinate Attention (CA), and Triplet Attention is the most common practice [80,106,107,108]. These mechanisms function as digital filters, forcing the neural network to “focus” computational resources exclusively on geometric features of the components, while simultaneously suppressing “noise” originating from the irrelevant background. At the same time, improved bidirectional feature pyramid networks, such as BiFPN and PANet, are extensively used for fusion of semantic information from different depth levels of the network, enhancing the ability to detect tiny targets such as bolts and pins [79].

#### 4.2.2. Defect Detection and Fault Classification

Detection of existing faults, such as broken porcelain insulator discs, rusted vibration dampers, and broken aluminum strands, is a technical problem considered more demanding and complex than the simple detection of components, mainly due to the minuscule size of defects relative to the total surface of the photograph, referred to as the small-object detection problem [109].

Modern literature highlights the development of specialized feature enhancement and alignment techniques, in which algorithms are trained not merely to recognize the shape but also to focus microscopically on texture distortions [29,83]. Since real, recorded fault data is by its nature rare due to the fact that network faults do not occur every day, researchers face class imbalance problems. To solve it, Few-Shot Learning methods are widely proposed, allowing the model to generalize and recognize a fault having “seen” only a finite number of times [84].

In addition, research is rapidly turning towards Semi-Supervised Learning [85] and Positive-Unlabeled (PU) Learning techniques [81]. These approaches allow the models to exploit the volume of “normal” (non-defective) and unlabeled images that drones collect daily, learning the concept of “normality” so as to detect any deviation as an “anomaly” [82,86].

#### 4.2.3. Vegetation Encroachment Monitoring

Uncontrolled vegetation (tree branches, shrubs) towards live conductors (vegetation encroachment) is, internationally, one of the main causes of short circuits (flashovers) and the ignition of catastrophic wildfires. Its monitoring is traditionally treated as a quintessential Semantic Segmentation problem [110].

Two-dimensional (2D) RGB images are mainly used, while encoder–decoder architectures such as variants of the U-Net network are applied [88]. These networks classify each individual pixel of the image into classes such as “vegetation”, “sky”, “ground”, or “cable”. However, the most reliable industrial approach to this specific problem is based on three-dimensional LiDAR data. In this context, specialized deep learning networks for point clouds, such as PointCNN, RandLA-Net, or architectures based on Point Transformers [87], directly classify each point (point-wise classification) in space. This three-dimensional approach is considered important, since it allows the mathematically precise calculation of the actual Euclidean distance between conductor and treetops, issuing valid warnings for preventive pruning [89].

#### 4.2.4. Thermal and Ultraviolet Inspection

As already mentioned, the analysis of thermal data has the exclusive aim of detecting “invisible” internal thermal anomalies that indicate electrical overload or internal material failures, e.g., breakdown in composite insulators [90]. Detection models, such as the thermal versions of YOLO, must necessarily be adapted to handle the low contrast and blurred boundaries of IR images. To achieve this, they often incorporate denoising algorithms in their initial stage.

Correspondingly, UV detection systems focus on visualizing corona-discharge phenomena, which indicate immediate current leakage into the air around an insulator. In these specialized cases, AI does not operate in isolation, but it is used as a “registration” tool. The algorithm matches the white/violet spot of the UV image with the precise geometric features of the insulator in the corresponding RGB image, providing engineers with a composite, comprehensible visual result.

#### 4.2.5. Safety-Distance and Sag Estimation with Three-Dimensional Methods (3D Sag Estimation)

Accurate estimation of the maximum vertical deflection of live conductors, due to their inevitable thermal expansion, especially during summer months under full load, requires 3D reconstruction of the scene.

Approaches that do not have LiDAR exploit Binocular Stereo Vision. In these systems, dual-lens cameras or pairs of images from a drone’s movement feed algorithms that perform key point matching, using methods such as Scale-Invariant Feature Transform (SIFT), Oriented FAST and rotated BRIEF (ORB), or advanced CNNs [92]. Parallax difference allows the computation of depth maps.

Alternatively, the most scientifically rigorous practice is carried out using filtering methods on LiDAR point clouds, e.g., voxel-based subsampling, for precise geometric separation of cables from environmental “noise”. Once the points belonging exclusively to the conductor have been isolated, mathematical catenary-curve-fitting models are applied, which simulate with millimeter accuracy the actual, physical geometry of the transmission line under its own weight, allowing the calculation of the precise sag [7].

#### 4.2.6. Multi-Task and Hybrid Models

One of the emerging trends at the cutting edge of research is the development of Multi-Task Learning Networks. These networks are designed to perform simultaneously more than one theoretically independent task through the same computational graph, saving computational resources.

For example, advanced networks such as AGMNet [94] are capable, with a single forward pass of the image, of detecting the precise location, within a bounding box, of a vibration damper and, at the same time, quantitatively classifying the degree of rust it bears.

Other hybrid, multi-level models incorporate image restoration processes internally, before the final detection stage. A characteristic example is the LARNet architecture [93], which takes a dark, rainy, or foggy image, performs the removal of “noisy” weather, e.g., deraining/dehazing, and then forwards the “cleaned” image to the YOLO detector. Such approaches guarantee extremely stable and seamless performance regardless of extreme weather conditions during a UAV’s flight [111]. Finally, the use of innovative models that combine Graph methods, such as Graph Convolutional Networks (GCNs), is also observed; these do not view the components in isolation, but take into account their “topological relationship” and interaction, e.g., an insulator is always connected to a pylon, effectively increasing detection accuracy under conditions of heavy visual occlusion [95].

### 4.3. Datasets, Metrics, and Evaluation

The success, reliability, and generalization capability of any Computer Vision system depend directly and absolutely on the quality, quantity, and diversity of the training data, as well as on the rigor of the metrics used to evaluate it. In what follows, the data landscape in the field of power line inspection is investigated.

#### 4.3.1. Publicly Available Datasets

One important finding from the conducted literature review is the lack of large, open, and globally accepted datasets. In contrast to other fields such as autonomous driving, where there are enormous benchmarks, such as COCO, KITTI, and Cityscapes, the energy industry is reluctant to release data due to strict security protocols.

Nevertheless, certain critical public datasets have appeared that function as reference benchmarks for academic research, including the following:

- CPLID (Chinese Power Line Insulator Dataset): Perhaps the most well-known public dataset, focused exclusively on insulators. It contains images of “healthy” insulators as well as defective insulators with broken discs. Although widely used, it is now considered small in size and relatively easy for state-of-the-art networks [112,113].
- TTPLA (Transmission Tower and Power Line Aerial images): A dataset for semantic-segmentation tasks. It contains thousands of high-resolution images with detailed pixel-level annotations for transmission cables and pylons, forming the basis for research on autonomous UAV navigation [24,36].
- SFID (Synthetic Foggy Insulator Dataset): A more specialized dataset designed to study the effect of fog. It is based on real insulator images to which realistic synthetic fog “noise” has been added [62].
- PLDU/PLDM (Power Line Dataset Urban/Mountain): Datasets focused exclusively on detection of transmission lines in urban and mountain environments, useful for testing performance against complex backgrounds.

Despite their usefulness, these public datasets are usually limited to very specific categories of components, e.g., insulators only, and exhibit a lack of samples with rare defects [114,115,116,117].

Beyond the established publicly available datasets, a newer generation of task-specific benchmark datasets has emerged in the most recent literature (2023–2025). Specifically, EPFD targets electric-fitting object detection [114], the RSIn-Dataset provides a dedicated UAV-based insulator detection benchmark [115], Stower-13 [116] and TTower-345 [117] target automatic classification and naming of transmission tower types, VEPL addresses vegetation-encroachment segmentation [89], and InsPLAD and its synthetic counterpart Power-Synth target component detection under weather-diverse conditions [35]. Several studies explicitly frame their proposed dataset as a response to the same gap, that although many public datasets are available, line patrol inspection datasets are scarce [115], while a shortage of comprehensive, publicly available datasets related to transmission tower inspections [116] and datasets having a significant number of examples of faults [14] has also been observed. Even the oldest and most widely reused resources in the field, such as PLDU and PLDM, remain narrowly scoped to visual power line detection, constituting the closest available approximation to a shared benchmark [118].

Note that evaluation benchmarking is also referred to as a problem, since broader comparisons and standardized benchmarks towards enhancing reproducibility and comparability are limited [105]. In [15], the authors conclude that at a field-wide level, the limitations of available datasets, quality of annotations, inconsistent validation practices, and gaps between model optimization metrics and real-world operational testing remain bottlenecks across the related literature. This fragmentation compounds the closed-ecosystem problem, further discussed in the following section, referring to the fact that the majority of top-performing models are trained and evaluated on private, non-disclosed data. Even where public benchmarks exist, they are task-specific and rarely used consistently across studies, making direct, fair comparison between competing methods difficult beyond a very limited number of narrow sub-problems. Therefore, the establishment of comprehensive, multi-task, publicly available benchmarks, spanning the full range of components, defect types, and platforms, together with a standardized evaluation protocol addressing the domain-generalization gap, remains an open issue.

#### 4.3.2. Private and Industrial Datasets

Due to the inadequacy of public datasets, the majority of the most advanced models presented in the literature (~70% of the 148 studies) are trained, validated, and tested on private datasets. These data are usually collected directly from national grid operators, such as China Southern Power Grid and networks in India, Europe, and North America, or from private companies specializing in aerial inspections.

Private datasets offer the advantage of absolute realism. They contain images from the real, everyday wear of the networks, under varying weather conditions and geographical terrain, spanning from sandy deserts to snow-covered mountains. However, their strict non-disclosure, whether due to national security concerns of critical infrastructure or corporate confidentiality, makes it absolutely impossible to directly and fairly compare the proposed algorithms among independent researchers, creating a “closed ecosystem” of research [25,119].

#### 4.3.3. Synthetic Datasets and Data Augmentation

To address deep NNs’ need for multiple data, as well as to solve the problem of a complete lack of photographs of rare defective components, researchers are increasingly turning to the generation of Synthetic Data.

A widespread practice is the use of graphics engines, such as Unreal Engine 4 and Unity, to create virtual, photorealistic environments. Within these worlds, 3D models of pylons and insulators are placed, allowing the automated extraction of thousands of images from every possible shooting angle and under every possible lighting condition, accompanied by accurate ground-truth annotations [8,120,121,122,123,124].

Another approach is based on the use of Generative Adversarial Networks (GANs), e.g., StarGAN-v2. GANs are used for “style transfer”: they take a real photograph of an insulator shot against a clear sky and mathematically generate the same photograph but under conditions of torrential rain, dense fog, or darkness [35]. This artificial “data augmentation” aims to force detection models to learn to ignore weather phenomena [125,126,127,128].

#### 4.3.4. Benchmarking Practices and Domain Shift

Traditionally, evaluation of models in the examined literature is carried out through the typical splitting of the data into training and test sets with an 80/20 ratio. However, the current practice suffers from a critical methodological gap: the problem of Domain Generalization.

Very few studies dare to evaluate their models in entirely different “domains”, performing cross-domain evaluation. For example, if a YOLO network is trained on images from a low-voltage urban network and is then asked to detect faults in a mountainous, ultra-high-voltage network in a forested area, its performance usually collapses, indicating domain shift problems. This lack of cross-validation limits confidence in the systems’ ability to operate on a global scale, beyond local networks on which they were trained.

Several field-tested techniques for mitigating this domain shift problem are in fact present in the reviewed literature, although they are not consistently framed as such. Transfer learning is the most well-known practice. The used model is fine-tuned while it is pre-trained on one operational setup using only a small fraction of labeled data from a new environment. Transfer learning has been reported to achieve up to 98% mean average precision on the new setup in [129], substantially reducing the data-collection burden associated with the deployment of the model on an unfamiliar network. Unsupervised domain adaptation offers a more principled alternative. Specifically, in [120], a cross-domain multilevel feature-alignment network trained to bridge synthetic and real-world insulator imagery improved AP50 by 8.6% over a source-domain-only baseline. Weather-specific-domain shift has also been directly targeted. In [58], synthetic rain, snow, and fog augmentation improved detection mAP by 2.4% under matching extreme-weather conditions while also improving clear-weather generalization by 1.2%, while in [130], a dedicated weather-domain synthesis network achieved 99.5% mAP@50 in cross-dataset evaluation under foggy conditions. Cross-task generalization has also been empirically demonstrated. In [100], a lightweight power line detector evaluated out-of-domain on the unrelated VisDrone benchmark achieved a 13.5% relative mAP improvement over its baseline while simultaneously reducing model size from 16.4 MB to 6 MB, indicating that architectural lightweighting and generalization capability are not necessarily in conflict.

Cross-regional federated learning is also represented in the examined literature. In [97], the authors train a shared detection model across multiple utility companies’ locally held power inspection datasets without centralizing the underlying images, reporting a federated model mAP of 0.95 compared to 0.424–0.618 for models trained on any single client’s data in isolation, and introduce a momentum-based aggregation scheme (FedRM) that achieves up to a 6.8-fold training-efficiency improvement over standard federated averaging. The authors, however, identify two practical barriers that limit real-world adoption of this approach, that should be considered from researchers in future works: (1) performance degrades when client data is highly non-identically distributed and the momentum parameter is set too aggressively, and (2) the framework requires an incentive mechanism to motivate otherwise-competing utility companies to participate in a shared training process, since the fusion is voluntary and lacks an inherent commercial motivation.

#### 4.3.5. Standard Computer Vision Metrics

For the rigorous quantification of performance, the examined studies use internationally established metrics, described in the following. Mean Average Precision (mAP) is the most frequently reported metric for object detection. It computes the average of the precision for each component class, taking into account the extent to which the predicted bounding box matches the actual object. In the examined literature, mAP performances greater than 90% are considered acceptable for industrial use.

Precision and Recall are other standard metrics. Precision measures how many of the “faults” detected by the system were actually faults. Recall measures the proportion of actual faults that the system ultimately managed to detect. Especially in defect detection, Recall is more critical, since missing a real fault can prove fatal. The F1-Score is the harmonic mean of these two.

For semantic-segmentation tasks, e.g., vegetation detection or cable extraction, the main evaluation metrics are the Intersection over Union (IoU) and the Dice coefficient, which measure with mathematical precision the percentage of overlap of the prediction’s pixels with the object’s actual mask.

#### 4.3.6. Specialized Application-Domain Metrics

Beyond standard computer science metrics, specific applications of the energy industry also require specialized indicators.

Root Mean Square Error (RMSE) is widely used to assess geometric deviation in physical measurements. For example, in LiDAR or stereo-vision systems, the deviation of the computed sag from the actual one in centimeters is measured, or the error in estimating the thickness of the ice that has accumulated on the cables [91,131].

For certain objects that require orientation, such as corona rings or the tilt angle of insulators, Angle Accuracy is measured based on Oriented Bounding Boxes (OBB).

#### 4.3.7. Industrial Key Performance Indicators (KPIs) and Hardware Constraints

From the strict viewpoint of industry and grid operators, theoretical accuracy in the laboratory is not considered sufficient for the adoption of a system. For this reason, most recent studies (2023–2026) increasingly focus on the following computational performance indicators:

- Processing Speed (FPS): The rate at which the model can process images on the drone. A system is considered suitable for “real-time” application only when it achieves a speed of at least 25–30 FPS [60,132].
- Model Size and Parameters: The total number of trainable parameters of the NN. State-of-the-art models are often shrunk below 10–15 MB, e.g., MobileNet or YOLOv8-tiny models [31], in order to fit into the small RAM of the drone’s boards.
- Computational Load (Giga Floating-Point Operations, GFLOPs): The number of floating-point operations. The smaller it is, the less energy the microprocessor consumes, which directly extends the autonomous UAV’s maximum flight time.

### 4.4. Synthesis of Results and Industrial Applications

A comprehensive, paper-by-paper comparison of the reviewed methods, covering advantages, disadvantages, deployment suitability, typical accuracy, complexity, and computational time for all 148 reviewed works, is provided in Table S1 in the Supplementary Materials. From this extensive Table, several comparative patterns emerge.

The most consistently cited strength across the examined literature is a favorable accuracy/efficiency trade-off achieved through architectural lightweighting. Lightweight YOLO variants [100] report designs that retain broad cross-domain applicability, while Transformer-based detectors such as PLD-DETR [133] achieve superior parameter utilization efficiency through multi-scale feature fusion. A smaller subset of studies reports strong label efficiency, with semi-supervised DETR variants [85] achieving competitive performance using only 10% labeled data, directly addressing the annotation bottleneck common to this domain.

Weather and domain sensitivity are consistently reported limitations, with several detectors explicitly noting degraded performance under adverse lighting or image-quality conditions [85]. A recurring limitation is also the computational cost incurred by architectural enhancements. Attention-augmented or two-stage detectors targeting difficult, densely packed defects report substantially higher GFLOPs than their single-stage counterparts [109], illustrating the same lightweight-versus-capability tension already discussed for YOLO- and Transformer-family detectors.

About deployment suitability, the large majority of studies (~36.5%) target UAV or drone-based platforms (54 of 148 studies explicitly specify UAV/drone deployment), with a further substantial subset (30 studies) targeting edge or embedded hardware such as Jetson-class boards. A smaller cohort (17 studies) is designed exclusively for offline or desktop-GPU execution, and a specialized group (6 studies) targets airborne or mobile LiDAR platforms rather than optical imagery. Notably, more than the rest of the studies, referring to almost 30% of the examined literature, do not specify a deployment target at all, an omission that compounds the reporting gap already discussed in Section 4.4.2.

Regarding typical accuracy, the highest-performing studies report precision, mAP, or AP values approaching or exceeding 99% under controlled evaluation conditions [66,113,127]. However, consistent with the dataset-bias trend detailed in Section 4.4.2, a substantial share of the corpus (56 of 148 studies) does not report a quantitative accuracy metric at all, limiting direct cross-study comparability even where methods are conceptually similar.

Reported model sizes, referring to model complexity, span more than two orders of magnitude, from sub-3-million-parameter lightweight detectors optimized for edge deployment [100,124,134] to Transformer- and attention-based heavy architectures exceeding 60–230 GFLOPs [85,109], reinforcing the architectural trade-off introduced in Section 4.4.1 for Transformer-based methods [12,133,135].

Finally, regarding computational time, the fastest reported detectors exceed 200 frames per second [97,108,128,134,136], meeting the real-time onboard-UAV requirements discussed in Section 4.4.3. In contrast, offline or LiDAR-based pipelines report processing times measured in seconds, e.g., segmentation-based aerial-image pipelines requiring over 400 s per image in [64] and LiDAR point-cloud scene processing requiring 8–12 s per scene in [6], highlighting that real-time deployment is achievable primarily for lightweight, single-stage, optically driven methods rather than for heavier or point-cloud-based approaches.

The value of academic research lies in its ability to constitute practical solutions for the real world. Therefore, in the following, this section presents a synthetic evaluation of the recorded results, as well as the current state of industrial adoption of Computer Vision systems.

#### 4.4.1. Model Performance Comparison

**Figure 8:**
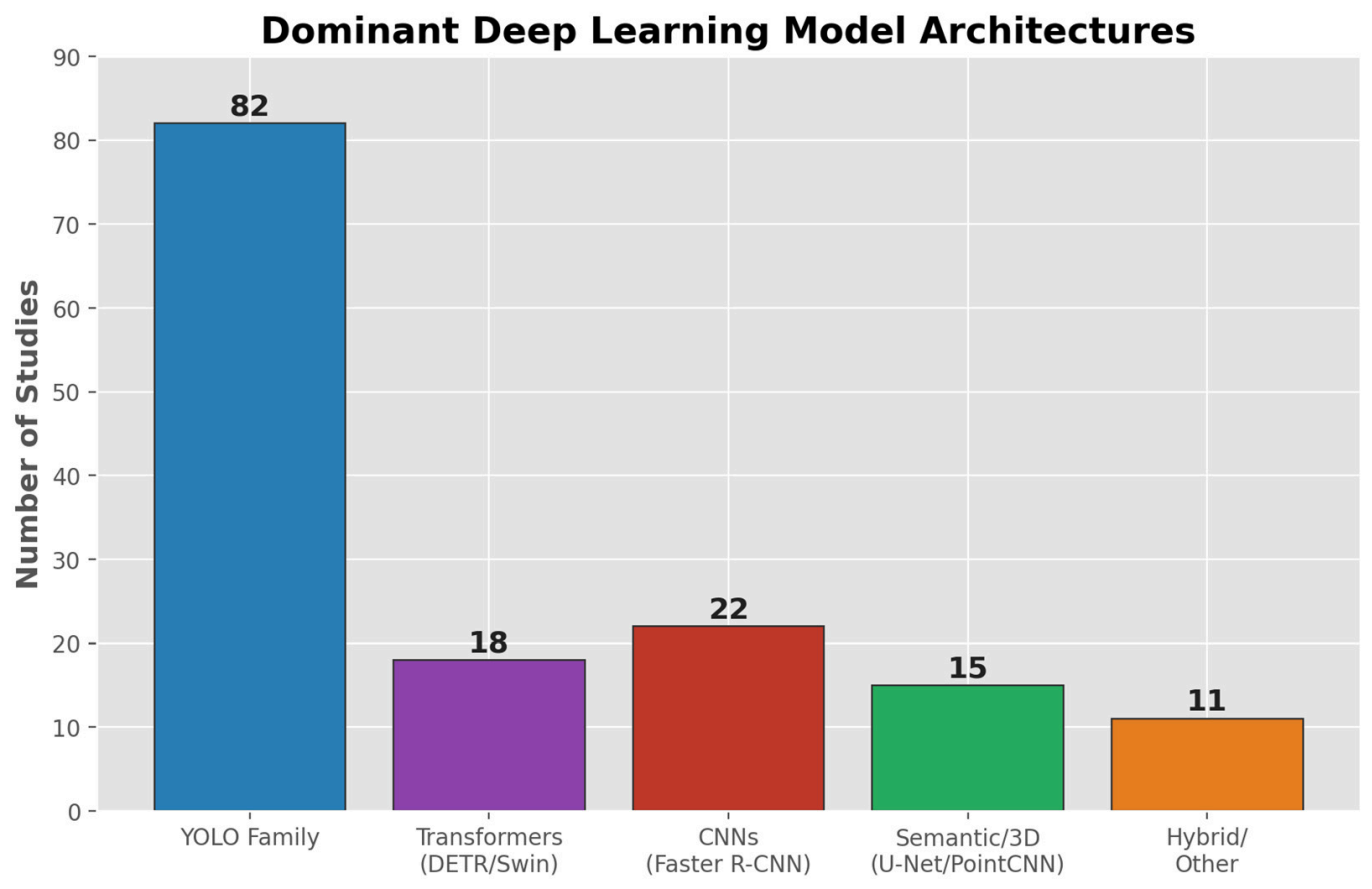
Comparative distribution of deep learning architectures. The analysis confirms the dominance of the YOLO model family due to its superiority in real-time edge computing applications, alongside the rapid increase in the use of Transformer architectures for detecting complex visual correlations.

The quantitative synthesis of the results demonstrates the dominance of YOLO models for the majority of real-time component-detection tasks. In particular, the most recent versions, such as YOLOv8, YOLOv10, and YOLOv11, consistently achieve mAP performances exceeding 90% on well-lit, clear aerial photographs. Their most significant advantage, however, lies in their architectural optimization that allows extremely fast inference. Techniques such as depthwise separable convolution enable these models to achieve “industrial-grade” speeds (30–60 FPS) even when running on small, low-power processors [28,33,104,136,137,138,139]. Figure 8 summarizes the comparative distribution of the deep learning architectures identified across the examined literature.

The dominance of YOLO models could be better explained through the fundamental architectural difference between single-stage and two-stage detectors. YOLO-family models directly regress bounding boxes and class probabilities from a single dense grid pass over the image, eliminating the separate region-proposal stage that two-stage detectors require. Across the reviewed literature, this design is consistently justified, offering reduced computational complexity, lower memory footprint, and compatibility with resource-constrained UAV and edge hardware [79,99,125,139,140,141].

This same design choice, however, introduces a well-documented trade-off between inference speed and missed tiny defects like cotter pin losses. The coarse spatial grid used for single-shot regression is inherently less sensitive to small, sparse targets than region-proposal-based alternatives. One field-wide benchmarking study [114] reports that even the best-performing detectors evaluated achieve an AP-for-small-objects (AP-small) of only 17.7% on electric-fitting components, explicitly identifying small-target detection as the primary factor that restricts the further improvement of models’ performance. This limitation is particularly consequential for defects such as missing or loosened cotter pins, which occupy an average of just 1.7% of image area and are easily suppressed below a model’s confidence threshold, as reported in [30]. Specifically, in [30], in order to reliably detect them required to abandon a standard single-stage pipeline in favor of a dedicated small-object architecture combining a ResNeSt backbone, Feature Pyramid Network, and Receptive Field Block, which improved sub-96 × 96-pixel detection accuracy by 10.45% over the unmodified baseline. Similar re-engineering is reported throughout the examined literature. In [140], dedicated small-object detection layers and attention modules raise AP-small from typical single-digit-to-low-double-digit baselines to 34.38% on the VisDrone benchmark and to 61.3% on one evaluation set while collapsing to 0% on another, more challenging one, and small, structurally fragile components such as bolts and pins are repeatedly singled out as the hardest category to recognize reliably [125]. The preference among researchers for single-stage detectors is largely driven by their speed. In [141], the authors use a single-stage model, reporting a nearly 30-fold inference speed increase over comparable two-stage detectors.

On the other hand, there are researchers who argue in favor of two-stage detectors. In [109], the authors perform a direct, class-level comparison on the same evaluation set, reporting that single-stage detectors YOLOv5 and YOLOv8 achieve only 46.4% and 65.3% AP@0.5 on the defect class, respectively, compared to 81.9% for their proposed two-stage model, at a cost of dropping from 79 FPS to 19 FPS. The same authors propose a two-tier deployment strategy as a practical resolution in [109]. Fast, single-stage models perform initial onboard triage on edge hardware, while slower, two-stage models perform secondary, higher-accuracy re-inspection of flagged images at ground-based operation and maintenance centers. This division of labor between real-time screening and offline verification represents a viable path to address the trade-off between field deployment speed requirements and the reliable detection of the smallest and often most safety-critical defects.

The last two years have seen an explosive rise in models based on the Transformer architecture, such as DETR and the Swin Transformer. These models, which were originally developed for natural-language processing, present a unique comparative advantage: the ability to detect long-range visual correlations (global contextual awareness). Instead of “seeing” only a small region of the image as classic CNNs do, Transformers perceive the entire geometry of the pylon simultaneously. This makes them more effective at detecting “hidden” defects in extremely complex backgrounds or in cases of severe object overlap. Their disadvantage, however, remains their dramatically high computational complexity, having an enormous number of parameters and GFLOPs, which limits their widespread use in real-time edge applications without prior heavy compression [12,133,135].

In the field of semantic segmentation of point clouds from LiDAR sensors, models based on graph-attention mechanisms, such as Point-based Deep Learning networks, now consistently achieve IoU performances above 85%, far surpassing traditional, heuristic filtering methods that required manual parameter tuning by the user [142].

#### 4.4.2. Dataset-Based Evaluation Trends

A deeper analysis of the results reveals a worrying trend: strong dataset bias. Specifically, many research groups present algorithms that record very high performances, e.g., mAP of almost 99%, when tested on their own, local, and private datasets. However, in studies that attempt cross-dataset validation, it is proven that these exact same models exhibit a rapid drop in performance, often below 60–70%, when used to operate on images from different infrastructures, e.g., moving from the network of China to that of India, or under conditions of extreme weather phenomena, such as heavy rain or fog.

This domain shift phenomenon reveals that, despite the impressive “laboratory” figures published, the fundamental problem of robustness and generalization has not been solved. The models tend to “memorize” and overfit to specific visual backgrounds instead of learning the universal, intrinsic characteristics of the component fault.

#### 4.4.3. Real-World Deployment Trade-Offs

The transition of an algorithm from a laboratory environment with powerful GPU clusters to the field, installed on drones “struggling” with strong winds, requires hard trade-offs. Engineers need to find the trade-offs between three conflicting objectives.

- Absolute Accuracy: The system’s ability to detect every fault (zero false negatives) without giving false alarms (zero false positives).
- Inference Speed: An extremely fast response is absolutely necessary. If the drone does not “perceive” the cable in time, it may collide with it. Furthermore, immediate processing allows operators to be notified in real time.
- Resource and Energy Consumption: Every watt of energy the microprocessor consumes is subtracted from the drone’s motors, reducing the useful flight time.

Discussions among the examined literature indicate that the practical solution lies in the “lightweighting” of models [32,140]. In industrial practice, it is considered entirely acceptable to sacrifice 1–2% of a model’s absolute accuracy if this compression allows it to run at 40–50 FPS. At this rate, the drone has time to capture and analyze multiple photographs of the same defected insulator from slightly different angles as it passes by, offsetting the initial, small loss of accuracy through the statistical confirmation provided by multiple shots [23,38,118,134,141,143,144,145,146,147].

Beyond algorithmic performance, a small subset of studies provides concrete economic statistics that begin to quantify these trade-offs in monetary terms, summarized in Table 3.

At the platform level, per-kilometer inspection costs vary substantially by modality: manned helicopter patrols are reported at over USD 100 [148] to EUR 266 [15] per kilometer, whereas UAV-based inspection reduces this cost by approximately 66% [15], with reported per-kilometer costs across drone, fixed-wing, and helicopter platforms ranging from EUR 60 to EUR 1300 depending on sensor payload and coverage requirements [89]. At the hardware level, onboard edge-computing platforms exhibit an order-of-magnitude price range, from approximately USD 75 for a Raspberry Pi 4B to USD 670 for a Jetson Orin Nano [59], directly linking the model-compression trade-offs discussed above to a concrete procurement decision.

A second, less visible cost dimension concerns data preparation and model development. Manual annotation remains a substantial labor bottleneck, with reported tagging rates of approximately 30–40 images per hour translating into hundreds of person-hours for a single training dataset [14,75], and pixel-level segmentation labels requiring approximately 15 times more annotation effort than bounding-box labels for the same imagery [118]. Tailoring a deep learning pipeline to a new operational environment can further require one to six months of dedicated Research and Development (R&D) effort [149].

Notably, one work [15] explicitly frames automation as economically justifiable only for frequent, high-impact defect types, cautioning that automating the detection of rare failure modes may not be cost-effective given these development costs. Overall, these findings indicate that, while the reviewed literature consistently frames UAV-based Computer Vision as cost-effective relative to manned inspection, this claim is rarely supported by a complete accounting of acquisition, annotation, and development costs. A rigorous Total Cost of Ownership or Return on Investment analysis comparing automated and traditional inspection pipelines remains absent from the literature and constitutes a concrete direction for future work.

Although most of the literature remains at the simulation level, several extensive studies, mainly from China and India, are accompanied by case studies of wide-scale application on real national power transmission networks [25,106]. These studies offer invaluable, practical data regarding the reduction of inspection time, efficiency at control centers, and targeted maintenance.

Specifically, the use of fully autonomous UAVs equipped with cameras and navigation algorithms reduces the on-site inspection time per pylon by 50% to 70% compared with traditional manual flights, since the drones fly in optimal trajectories without hesitation. Moreover, the use of artificial intelligence in central systems reduces the time spent reviewing thousands of aerial photographs by 80%. Instead of engineers spending multiple hours looking at clean components, the system “filters” the photographs and presents to them only those that contain defective indications, along with proposed bounding boxes of the faults. Finally, automated, geographically precise fault reporting leads to perfectly targeted interventions by maintenance crews. Knowing in advance the exact type of fault, e.g., a broken third insulator disc on pylon #145, technicians travel to the site already carrying the correct spare parts, minimizing power-outage time.

These generalized efficiency practices are documented by individual field-deployment reports across the reviewed literature, although hard, standardized operational metrics remain scarce. At the fleet scale, one of the largest reported industrial deployments comprises approximately 20,900 UAVs operated by a national grid operator for inspecting transmission lines rated at and above 66 kV [149], while an independent techno-economic comparison estimates that UAV-based inspection reduces the normalized cost per kilometer by approximately 66% relative to traditional ground- and helicopter-based methods, with reported per-kilometer costs across inspection modalities ranging more broadly from EUR 60 to EUR 1300 depending on platform and coverage [15,89]. At the level of individual defect-detection performance in the field, one smart-camera deployment across 37 substations correctly identified 92.2% of 295 manually confirmed defective assets when benchmarked against expert infrared-camera inspection, as reported in [15], and a LiDAR-based vegetation-hazard survey covering 160 km of transmission corridor in mountainous terrain identified and reported 77 vegetation-related hazards to the local grid operator, including one classified as an emergency-level hazard. Flight-level operational parameters are similarly reported in isolated cases. The field-tested tracking system presented in [47] maintains a flight cadence of approximately 6 s per 4 m of traversed line with a 3 s per-frame processing latency on a live 220 kV tower, while another field study [148] on live 110 kV and 345 kV lines reports a flight endurance of 25 min at an average speed of 11 km/h and empirically determined safe standoff distances of 5–7 m and 25–30 m, respectively [148].

Notably, however, none of the reviewed studies, including those cited above, provide the key operational metrics that are needed to validate their claims about real-world inspection efficiency, such as false-positive or false-negative reduction rates normalized per 100 pylons or per unit length of inspected line. The 50–70% inspection-time reduction and 80% photograph-review-time reduction reported at an industry-wide level [25,106] are, therefore, not yet traceable to a standardized, per-asset error-rate metric that would allow independent verification or cross-study comparison. The establishment of such a metric remains an open methodological gap for the field under study.

Despite these impressive benefits, the conclusions of the studies are clear: the modern energy industry still uses AI solely as an advanced “decision-support” tool for the human inspector (Human-in-the-Loop paradigm), while it is not used as a means of fully autonomous critical decision-making. The algorithm can detect the fault and propose a power cut, but the final confirmation of the fault and the decision to shut down a critical high-voltage line are always made by an authorized engineer, due to the enormous legal, financial, and insurance consequences that an error entails.

## 5. Challenges and Future Directions

**Figure 9:**
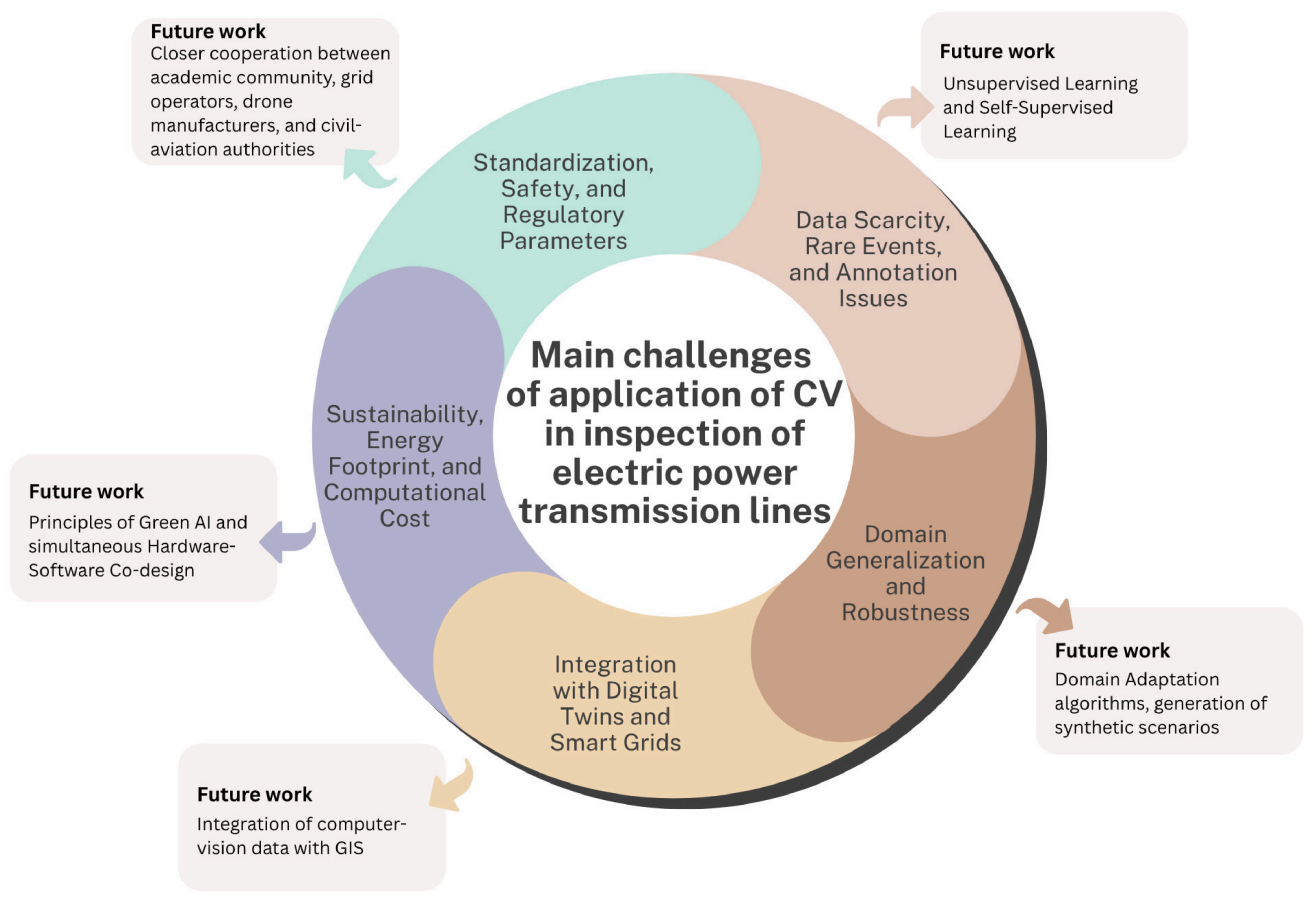
Challenges of the application of Computer Vision in everyday inspection of electric power transmission lines and suggested future research directions.

Despite the technological progress recorded in the examined literature, the full and universal application of Computer Vision in everyday inspection of electric power transmission lines still faces certain structural and systemic challenges. Successfully overcoming them will be a decisive turning point for the transition to the next generation of fully autonomous, “smart” power grids. Challenges along with future research directions are summarized in Figure 9.

The greatest obstacle, perhaps, to the development of truly infallible and powerful AΙ models remains the lack of high-quality data. While the industry possesses and daily collects millions of images of “healthy” and intact components, images of real, critical faults are extremely rare, resulting in data imbalance. An NN trained on a dataset in which 99% of the insulators are healthy tends to create statistical bias in favor of “normality”, often ignoring the rare defects. In addition, the process of image annotation is exhaustive. Especially for tasks such as semantic segmentation, where the precise manual coloring of each individual pixel of a cable is required, the process demands multiple hours of work by specialized engineers, due to the fact that a simple human annotator without a technical background cannot distinguish an oxidized damper [150]. The latter critically increases development-related costs. Therefore, future research must turn towards Unsupervised Learning and Self-Supervised Learning. In these architectures, the systems are trained directly on raw, unlabeled data, learning the internal structure of images without the need for human intervention [148,151,152].

As already mentioned, most modern models suffer from a lack of generalization capabilities. A model trained exclusively on the network of Greece, with the characteristic Mediterranean landscape, e.g., dry grass, intense sun, specific types of pylons, is expected to underperform or fail if used to recognize pylons in the snow-covered landscapes or dense forests of Sweden. Thus, there is an imperative need to develop specialized Domain Adaptation algorithms [57,105,130]. These algorithms allow a model to “transfer” the knowledge acquired in one region to a new, entirely unknown geographical region, using minimal new data. In parallel, the widespread use of GANs to generate synthetic scenarios of extreme rain, dense fog, and snowstorms is necessary in order to “harden” the networks against natural optical interference [35].

Future industrial inspection needs to go further than simple recognition of an individual defect. On the contrary, the goal is the immediate integration of this finding into a complete, holistic, and three-dimensional digital representation of the entire network. The close interconnection and integration of Computer Vision data, such as RGB photographs from UAVs and point clouds from LiDAR, with Geographic Information Systems (GIS) allows the creation of Digital Twins (DTs) [153]. Through a DT, grid operators do not simply see that “insulator A has a crack”. They can run complex physics-based simulations on the digital model to predict exactly how this crack will affect the overall stability of the pylon in case winds of 100 km/h blow in the area. Computer Vision thus becomes the “sensor” that continuously feeds the DT with real-time data.

The continuing trend toward building and training ever larger and deeper NNs, such as Vision Transformers, requires reasonable computational power and, consequently, great amounts of electricity in data centers [154]. This creates a paradox: the electric power industry uses extremely energy-intensive models to protect its own network.

On the other hand, running these models “on-board”, e.g., on edge hardware of a small drone, presupposes extremely low energy consumption so that the battery does not drain quickly and the useful flight time is not reduced. The concept of Green AI and the interdisciplinary field of simultaneous Hardware–Software Co-design are critical future research areas for the creation of sustainable solutions [37]. Thus, algorithms must be designed from the outset with the specific architecture of the microprocessor that will “run” them in mind.

Finally, the lack of international, rigorous standards for what level of accuracy of an AI system is considered “industrially acceptable” significantly delays widespread commercial exploitation. Moreover, the legislative framework in most countries remains extremely strict regarding autonomous UAV flights beyond the operator’s visual line of sight (Beyond Visual Line of Sight, BVLOS), especially when these flights take place within breathing distance of dangerous ultra-high-voltage (400 kV) equipment. Closer and ongoing cooperation is required among the academic community, grid operators, drone manufacturers, and national civil aviation authorities to establish realistic and safe regulations.

Overall, this work demonstrated that Computer Vision technology has radically redefined the way the world’s electric power infrastructure is inspected, maintained, and managed. Deep learning models installed on microprocessors aboard commercial drones can now detect tiny defects in critical components, such as porcelain insulators and dampers, with confirmed accuracy exceeding 90–95% [155], while at the same time offering remarkable real-time edge-computing capabilities during flight. In addition, the systematic use of three-dimensional point clouds via LiDAR technology ensures accurate monitoring of dangerous vegetation encroachment, while the innovative fusion of data from different sensors, such as thermal and visible-spectrum, addresses the limitations of natural lighting and weather conditions [30,75,105,129,149,156,157,158,159,160,161,162].

## 6. Conclusions

In this work, a review is conducted aiming to map the field of Computer Vision applications in electric power transmission line inspection for the period 2018–2026, analyzing 148 selected scientific studies.

For the global research community, the findings of this review demonstrate an imperative need to shift focus away from the perpetual creation of complex, heavy, and energy-intensive laboratory models. Researchers must turn towards the development of large, open industrial benchmarks, improved domain-adaptation algorithms, and resolution of fault-data-scarcity problems through self-supervised learning.

The conducted review identifies an ever-increasing use of UAVs equipped primarily with visible-spectrum cameras, while the “backbone” of most approaches relies on deep Object Detection NN. Particularly pronounced is the dominance of the YOLO model family, justified by its ability to offer an ideal trade-off between high mAP and fast inference speed. Moreover, since the ultimate goal is for these systems to operate in real-time and fully autonomously on edge devices with limited resources, researchers have focused heavily on developing their lightweight variants. To offset the inevitable loss of accuracy caused by reducing the network’s parameters, they employ innovative attention mechanisms focusing on the important features of components, ignoring complex backgrounds. At the same time, an emerging shift toward Transformer-based models is observed, offering enhanced global contextual awareness of images and handling occlusion problems efficiently, although they usually require increased computational power.

For industrial practice and national grid operators, immediate adoption of CV-based technologies is necessary, as it offers immediate economic and operational benefits through the related reduction of inspection time, optimization of preventive maintenance, and elimination of risks for overhead line technicians. The final transition, however, to Smart Grids of the future requires the maturation of legislative frameworks for autonomous flights and overcoming “data silos” problems through open-access initiatives or Federated Learning among energy companies. Computer Vision in infrastructure inspection could, therefore, become the most fundamental technological pillar for energy security, sustainability, and resilience of future societies.

## Figures and Tables

**Table 1:** Queries executed in Scopus.

No.	Thematic Area	Explicit Scopus Query
Q1	Powerline Monitoring Platforms	(TITLE-ABS-KEY ((“power line” OR “transmission line” OR “overhead line”) AND (inspection OR monitoring) AND (UAV OR drone OR satellite OR vehicle)))
Q2	Sensor Technologies	(TITLE-ABS-KEY ((“power line” OR “transmission line” OR “overhead line”) AND (inspection OR monitoring) AND (“RGB camera” OR “thermal camera” OR LiDAR OR multispectral OR hyperspectral)))
Q3	Computer Vision Approaches	(TITLE-ABS-KEY ((“computer vision” OR “machine vision” OR “image processing” OR “deep learning”) AND (“power line” OR “transmission line” OR “overhead line”) AND (inspection OR monitoring) AND (“object detection” OR “defect detection” OR classification OR segmentation)))
Q4	Datasets	(TITLE-ABS-KEY ((“power line” OR “transmission line” OR “overhead line”) AND (inspection OR monitoring) AND (dataset OR database)))
Q5	Evaluation metrics	(TITLE-ABS-KEY ((“power line” OR “transmission line” OR “overhead line”) AND (inspection OR monitoring) AND (evaluation OR performance OR “evaluation metrics” OR “performance metrics”)))
Q6	Defect-detection performance	(TITLE-ABS-KEY ((“power line” OR “transmission line” OR “overhead line”) AND (inspection OR monitoring) AND (“defect detection”) AND (evaluation OR performance OR “evaluation metrics” OR “performance metrics”)))

**Table 2:** Summary of Computer Vision tasks in electric power transmission line inspection, along with the prevailing sensors, dominant methods, key evaluation metrics, and representative studies.

Vision Task	Primary Sensors	Dominant Methods/Architectures	Key Metrics	Representative Studies
Component detection and classification	RGB (UAV)	YOLOv3–v11, RT-DETR; attention (CBAM, CA); BiFPN/PANet	mAP, Precision, Recall, FPS	[76,77,78,79,80]
Defect/fault detection	RGB, IR	Lightweight YOLO; feature alignment; Few-Shot, Semi-/PU-Learning; anomaly detection	mAP, Recall, F1	[29,81,82,83,84,85,86]
Vegetation encroachment	RGB, LiDAR	U-Net (2D); PointCNN, RandLA-Net, Point Transformer (3D)	IoU, Dice, distance error	[87,88,89]
Thermal/UV inspection	IR, UV	Thermal-adapted YOLO; denoising; RGB–UV registration	mAP, hotspot detection	[5,26,64,65,66,90]
Sag/safety-distance (3D)	LiDAR, stereo RGB	Voxel filtering + catenary fitting; binocular stereo; keypoint matching	RMSE (cm)	[7,91,92]
Multi-task/hybrid	RGB (+IR)	AGMNet; LARNet (restoration + detection); GCNs	mAP + task-specific	[93,94,95]

**Table 3:** Reported costs across the reviewed literature, spanning platform-level inspection costs, hardware procurement, and hidden data-preparation/development costs.

Ref.	Cost Item	Cost Item	Reported Value	Context/Comparison
[148]	Manned helicopter inspection cost	Manned helicopter inspection cost	>USD 100/km	Traditional inspection cost baseline
[15]	UAV inspection cost reduction	UAV inspection cost reduction	≈66% costs reduction	Relative to a normalized EUR 266/km traditional baseline
[89]	Per-kilometer cost by platform	Per-kilometer cost by platform	EUR 60–1300/km	Range across drone, fixed-wing, and helicopter platforms
[59]	Edge-computing hardware cost	Edge-computing hardware cost	USD 75–670	Raspberry Pi 4B up to Jetson Orin Nano
[14,75]	Manual annotation rate	Manual annotation rate	30–40 images/hour	Translates to hundreds of person-hours per training dataset
[118]	Segmentation vs. bounding-box labor	Segmentation vs. bounding-box labor	≈15× more effort	Pixel-level labels vs. bounding-box labels, same imagery
[149]	Pipeline retargeting R&D time	Pipeline retargeting R&D time	1–6 months	Adapting a trained model to a new operational environment

## Data Availability

No new data were created or analyzed in this study. Data sharing is not applicable to this article.

## Supplementary Materials

The following supporting information can be downloaded at: https://www.mdpi.com/article/10.3390/s26154819/s1, Table S1: Comparative aspects of the 148 documents included in the literature review: advantages, disadvantages, deployment suitability, typical accuracy, complexity, and computational time. All references included in the Table are also included in the main document.

## Abbreviations

The following abbreviations are used in this manuscript:

UAVs	Unmanned Aerial Vehicles
YOLO	You Only Look Once
RES	Renewable energy sources
RGB	Red/green/blue
LiDAR	Light Detection and Ranging
AI	Artificial Intelligence
CNNs	Convolutional Neural Networks
DETR	DEtection TRansformer
CV	Computer Vision
PRISMA	Preferred Reporting Items for Systematic Reviews and Meta-Analyses
IR	Infrared
KPIs	Key Performance Indicators
GANs	Generative Adversarial Networks
BVLOS	Beyond Visual Line of Sight
AAVs	Autonomous Aerial Vehicles
NN	Neural network
FPS	Frames per second
UV	Ultraviolet
SNR	Signal-to-noise ratio
ACCC	Aluminum-conductor composite-core
CBAM	Convolutional Block Attention Module
CA	Coordinate Attention
PU	Positive-Unlabeled
2D	Two-dimensional
3D	Three-Dimensional
SIFT	Scale-Invariant Feature Transform
ORB	Oriented FAST and rotated BRIEF
GCNs	Graph Convolutional Networks
CPLID	Chinese Power Line Insulator Dataset
TTPLA	Transmission Tower and Power Line Aerial images
SFID	Synthetic Foggy Insulator Dataset
PLDU	Power Line Dataset Urban
PLDM	Power Line Dataset Mountain
AP	Average Precision
mAP	Mean Average Precision
IoU	Intersection over Union
RMSE	Root Mean Square Error
OBB	Oriented Bounding Boxes
GIS	Geographic Information Systems
DTs	Digital Twins
SSD	Single Shot MultiBox Detector
SVM	Support Vector Machine
AP-small	AP-for-small-objects
R&D	Research and Development
