# Supplementary material for: Advances in Computer Vision and Sensor-Based Methods for Intelligent Power Transmission Line Inspection
Source: Sensors (Basel). 2026 Jul 29;26(15):4819. doi: 10.3390/s26154819 (PMC13468763; doi:10.3390/s26154819)
Supplement: Supplementary file 1 [file sensors-26-04819-s001.zip › sensors-4412894-supplementary.pdf]

Supplementary Materials

Table S1. Comparative aspects of the 148 documents included in the literature review: advantages, disadvantages, deployment suitability, typical accuracy, complexity, and computational time. All references included in the Table are also included in the main document.

Ref.	Advantages	Disadvantages	Deployment suitability	Typical accuracy	Complexity	Computational time
[1]	Eliminates background noise, exceptional efficiency–accuracy balance, multi-view feature enhancement using 3D models.	Potential for further lightweight optimization; intricacy of 3D model development.	UAVs/edge devices	mAP@50: 0.9811 ± 0.0057, F1-score: 0.9805 ± 0.0053, AP: 0.7249 ± 0.125	2.1M parameters, 6.4G FLOPs (also reported as 5.5M parameters, 10.4 GFLOPs)	Not reported
[2]	End-to-end architecture effectively modeling spatial correlations, eliminates additional computations, enriches contextual information.	Degraded performance in dark/foggy conditions; fails frequently from top-down camera angles; heavily reliant on aerial photograph quality.	UAV / desktop GPU	Not reported	ResNet-101 backbone	Not reported
[3]	Lightweight and robust; handles multiple defects (broken sheds, balisor fading, broken wires, rust); fast performance due to custom Sobel filters.	Low precision for split detection due to shadows, surface deformations, and complex illumination; hand-crafted features prone to failure.	Drone / NVIDIA Jetson TX2 GPU	Balisor fading defect detector: 100% recall, 76% precision	Darknet CNN framework	Not reported

[4]	Reduced acquisition cost via automatic synthetic datasets; stable time/space and depth differences from unique FPTLIR collection mode.	Not reported	Not reported	Not reported	YOLOv5s based	Not reported
[5]	High accuracy combined with lightweight structure; parameter count drastically reduced; captures multiple types of insulator defects.	Limited performance in adverse weather conditions; requires further optimization for extremely constrained edge devices.	Drones/edge computing devices	mAP@0.5 increased by 4.7%, Recall increased by 2.6%, mAP@0.5:0.95 increased by 1.4%	Parameter count reduced by 59.38%	Not reported
[6]	Improves accuracy while maintaining a lightweight effect; reduces information loss; extracts abstract features at different levels.	Increased model complexity over YOLOv8n; FPS is decreased; requires validation for other tasks.	UAV / desktop GPU	mAP50: 72.2%	Parameters: 2.8 M, GFLOPs: 8.9	Not reported
[7]	Overcomes spatial and safety constraints of multi-split phase conductors; suitable for unsupervised training.	Unknown effectiveness on quad split-phase conductors and electrical fittings other than strain clamps.	Dual UAVs / airborne edge-intelligent terminals	mAP@50: 60.9%, mAP@50:95: 30.6%, F1-score: 60.9%	Not reported	89 frames per second
[8]	Solves the problem of insulator multi-fault detection with a systematic analysis.	Not reported	Aerial images	Precision: 96.30%, Recall: 93.30%	Not reported	Not reported
[9]	Effectively learns label correlation and important structural features.	Small target and single feature of bolts make recognition hard; it needs improvement for end-to-end detection.	Not reported	2.9% increment in label-level accuracy, 6.5% increment in image-level accuracy	Not reported	Not reported

[10]	Reduces redundant computing by extracting candidate regions from segmented areas; high precision and lightweight design.	Misses extremely thin broken strands; interference from complex backgrounds, towers, and fittings causes false detection.	UAVs	Precision: 0.833, Recall: 0.769, F1-Score: 0.800	Not reported	11.5 Images/s
[11]	Addresses efficiency limitations; covers keypoint and skeleton attitude detection for multiple tower types; good generalization capability.	Not reported	UAVs	Not reported	Not reported	Not reported
[12]	Highly generalized and reliable; easily adaptable to new setups with limited training data; robust feature extraction.	Validation predominantly utilized low-voltage lines with single bells.	Not reported	98% mean average precision	Not reported	Not reported
[13]	Enhances feature differentiation; avoids excessive sample relationship differentiation; robust in complex backgrounds.	Max pooling loses critical target details; global attention obscures fine-grained differences; YOLO-L underperforms due to overfitting.	Resource-constrained environments	mAP: 77.7%	Not reported	Not reported
[14]	High-precision defect identification in complex environments; effective for small-scale missing-cap insulators.	Difficulty in collecting enough samples of insulator defects for training.	UAV/mobile devices	Not reported	Not reported	Not reported
[15]	Significantly improves real-time performance and detection accuracy while reducing model size and computational complexity.	Verified only on single defect type (self-exploding insulators); generalization compromised when pruning speed is too high.	Unattended UAV systems/edge servers	Accuracy: 97.4%, mAP@50 increased by 1.9%, mAP@50:95 increased by 5.9%	Parameters reduced by 23.5% (0.708M), computation reduced by	Not reported

					9.6% (0.8 GFLOPS)	
[16]	Fast collision detection using SAT; strong noise resistance and robustness; accurately simulates complete topology.	Extraction accuracy decreases when power lines are sparse or congested; it cannot meet real-time requirements due to global searches.	Vehicle-borne mobile laser scanning (MLS)	Recall rate: 98.28%, precision rate: 98.71%, RMSE < 0.04 m	Not reported	Not reported
[17]	End-to-end integration of segmentation; performs effectively on different types of power lines and towers.	Relies on many manual thresholds; lacks adaptiveness to varied regions or different sensors.	UAV LiDAR	Mean interaction over union: 82.73%, overall accuracy: 91.76%	Not reported	Not reported
[18]	Continuously optimizes feature space through automated credibility assessment; superior accuracy and precision metrics.	Static pre-selection of foreground and background regions for compressing feature vectors limits generalization.	Not reported	Average precision: 89.9%, average rejection rate: 13.7%, average false positive rate: 7.2%	Not reported	Not reported
[19]	Estimates PoD and VoD accounting for environment/load conditions; generates new features to overcome the lack of distinguishing traits.	A limited amount of fault data restricts coverage; augmented data only reflects existing fault characteristics.	Not reported	Not reported	Not reported	Not reported
[20]	Commendable real-time performance; highly adaptable to intricate and challenging environments.	Improvement in overall image accuracy is limited because power line targets occupy a relatively small proportion of the image.	Not reported	IoU: 74.79%, Prec: 90.21%, Rec: 81.40%, F1: 85.58%, Acc: 99.43%	Param: 27.80 M	29 FPS

[21]	Reconciles instance and class sampling; effectively handles complex, highly overlapping distributions and multiple pylon types.	Graph-based inference postprocessing has high computational cost; struggles with missing LiDAR point cloud data.	UAV LiDAR	Average precision (PR), recall (RE), and F1-scores of up to 95%	Not reported	Not reported
[22]	Well-balanced trade-off between accuracy and speed; achieves high performance using only 10% labeled data.	Performance influenced by weather and image quality; limited generalization to unstudied fault categories.	Real-time applications/ edge devices	mAP: 77.7, AP50: 75.0, Precision: 91.7, F1: 90.3, Recall: 88.9	67 M Parameters, 234 GFLOPs	85 FPS
[23]	Parameter-efficient tuning leads to substantial reductions in computation and storage costs.	Not reported	Not reported	Not reported	Not reported	Not reported
[24]	Strikes an optimal balance between accuracy, model size, and processing speed.	Not reported	On-board detection systems	mAP: 83.8%	Not reported	243 fps
[25]	Refines feature representation; robust performance in complex backgrounds and lighting changes; highly efficient.	Constrained in detecting small, intricate defects; lacks robustness across diverse weather scenarios.	UAVs	Average accuracy: 83.14%	Not reported	65 frames per second (65 ms)
[26]	Dynamically adjusts kernel weights based on spatial layout; resolves uneven density distribution; incorporates global contextual relations.	Global attention calculations consume excessive GPU memory, impacting model efficiency.	Airborne LiDAR	Average F1 score: 71.5%, overall accuracy (OA): 83.2%	Not reported	Not reported
[27]	Gradually refines decision boundaries; effectively eliminates noise introduced by pseudo-labeling.	Not reported	Edge	Not reported	Not reported	Not reported

[28]	Consistent performance with low variability; isolates ROI efficiently; enables accurate 3D ice thickness measurements.	Grouping limits detection of features with identical distances; it struggles with smooth structures and drastic lighting/texture changes.	Not reported	Average precision: 90%, F1 score: 86%, Average recall: 82%	Not reported	0.01 seconds
[29]	Significantly improves computational efficiency and target detection accuracy while remaining lightweight.	Low recall at very low false alarm rates; new modules increase inference time by 10-20%.	Not reported	mAP@0.5: 92.30%	10.2 M parameters, 25.5 GFLOPs	Not reported
[30]	Enhances small object detection; reduces parameter count by 46%; accelerates inference speed by 36%.	High-resolution S2 feature layer initially raised FLOPs before balancing strategies were applied.	UAVs/edge computing	mAP@0.5: 93.6%	Parameters reduced by 46% (52.8% reduction noted in backbone)	Inference speed accelerated by 36%
[31]	Not reported	Not reported	Not reported	Not reported	Not reported	Not reported
[32]	Hardware-friendly for embedded devices; effectively solves class imbalances and improves detection of difficult samples.	Not reported	Embedded intelligent edge devices	Not reported	Not reported	Not reported
[33]	Significantly reduces feature information loss of small targets; mitigates background interference leveraging contextual information.	Disadvantage of having a large parameter count (complex structure).	Not reported	P: 96.39%, R: 92.53%, F1: 0.94, mAP: 98.40% (Also reported F1: 0.94, mAP: 97.36%)	Highest parameter count	Not reported
[34]	Effectively extracts temperature curves; significantly reduces manual interpretation workloads; strong generalization capabilities.	Not reported	UAVs	mAP50: 99.4%, mAP50:95: 94.0%, Precision: 98.9%, Recall: 99.1%	40.5 M Parameters	Not reported

[35]	Compresses model size by 50% using group-level pruning; improves mAP0.5 over mainstream pruning techniques.	Parameter pruning can sometimes lead to a reduction in the efficiency of convolutional operations.	Not reported	Precision: 96.7%, Recall: 92.1%	Computational complexity reduced by 56.9% to 6.2 GFLOPs	Not reported
[36]	Automates detection and removes false/missed detections associated with visual fatigue in human inspections.	Not reported	Not reported	APval: 60.0%, AP50: 33.3%, APM: 58.0%, APL: 67.3%	5.82M Parameters	54 FPS
[37]	Eliminates NMS processing; drastically reduces detection time and meets real-time monitoring demands.	Not reported	UAVs	mAP: 91.65%	Not reported	55 FPS (F/s)
[38]	Reduces parameters and computational complexity; fully preserves channel-dimensional information; enhances small-target detection.	Replacing layers with Ghost-Conv and Bottlenecks incurs higher memory access overhead, resulting in longer inference times on GPUs.	UAVs / embedded devices / edge-computing	mAP: 86.73%	Not reported	38.1 FPS
[39]	Exploits complementary characteristics of VL and IR images; FFM requires a constant, small number of additional parameters.	Not reported	Drone	Not reported	12.5 million parameters	Not reported
[40]	Not reported	Not reported	Not reported	Not reported	Not reported	Not reported

[41]	High efficiency processing large datasets; robust across diverse conditions without requiring supplemental data (like trajectory).	Ground filtering over-segments ground from objects distant from the scanner; struggles with low-density data requiring re-parameterization.	Terrestrial laser scanning (TLS)/Mobile laser scanning (MLS)	F1 scores of 88.87–95.47%	Not reported	0.81 and 1.46 million points/sec
[42]	Automates deep learning model optimization and updating processes; superior feature extraction capabilities.	Extreme parameter sizes prevent single-server training; classical AutoML performs poorly in scenario applications due to data drift.	Cloud/Edge	Not reported	Not reported	Not reported
[43]	High detection efficiency effectively speeds up performance compared to traditional methods.	Automation and interactivity still need further improvement to reduce the failure rate in actual use.	Not reported	Not reported	Not reported	1.68s/m
[44]	Commendable real-time performance; highly adaptable and effective in intricate and challenging environments.	Not reported	Not reported	Not reported	Not reported	Not reported
[45]	Not reported	Not reported	Not reported	Not reported	Not reported	Not reported
[46]	Not reported	False detection occurs in regions similar to fault appearance; fails on faults hidden in cross-arms, shadows, or at long-range perspectives.	Not reported	Not reported	Not reported	Not reported

[47]	Consistently outperforms other methods in both power line region identification and risk object detection.	Subject to confusion from brightness contrast caused by sunlight or from generic wires that look like power lines.	Not reported	AP: 100%	Not reported	Not reported
[48]	Dynamically balances workloads across UAV/edge/cloud; handles continuous action spaces; minimizes task execution time.	Lacks incorporation of UAV mobility patterns and fault-tolerant mechanisms for adverse conditions.	Cloud-edge-end collaboration (UAVs, edge servers, cloud)	Not reported	Not reported	Reduces task execution time by up to 22% (Inference time on edge server ≈ 53ms)
[49]	Not reported	Not reported	Not reported	Not reported	Not reported	Not reported
[50]	Saves computational overhead by introducing group convolution.	Number of parameters increases significantly with augmentation of the convolution kernel size.	Not reported	Not reported	Not reported	Not reported
[51]	Smaller model size, lower computational complexity, higher inference speed, suitable for edge computing devices, balances accuracy, efficiency, and computational cost.	LUFDH has a slightly lower FPS than decoupled-head	AAVs, edge computing devices	P: 94.6%, R: 99.4%, mAP: 88.6%	3177336 Params, 13.5 GFLOPs	90 FPS
[52]	Addresses domain adaptation, evaluates the impact of artificial samples, improved alignment.	Not reported	Not reported	Not reported	Not reported	Not reported
[53]	Improves fault detection mapping, utilizes dynamic multi-scale convolution and attention mechanism, handles 9 fault types.	Not reported	Aerial transmission line inspection, Windows 11 with RTX 4060 GPU	map@0.5: 93.8%, map@0.5:0.95: 70.3%	Not reported	0.0079 s

[54]	Pixelwise power line detection, outperforms multiple baselines like DeepLabV3, PSPNet, and U-Net.	Introduction of the RRCNN branch greatly increases model complexity to bring more model parameters.	Aerial images	Not reported	Not reported	Not reported
[55]	Real-time defect detection of dampers, attention-guided.	Not reported	Not reported	Not reported	Not reported	Not reported
[56]	Multi-class EPTN fault detection model, avoids complex feature engineering, scalable, utilizes FPN for small aspect ratios.	Initial training took ~50h on a local CPU, excessively time-consuming without transfer learning.	UAV images, Google Colab (2 Xeon CPUs, Tesla K80 GPU)	Precision: 100.00%, 97.22%, 98.27%; Recall: 78.67%, 67.34%, 73.94%, 83.56%; F1-score: 87.41%, 80.49%, 84.00%, 89.71%	Not reported	Not reported
[57]	Decreases false-negative rate, works well on objects smaller than 96x96 px.	Not reported	UAV images, Intel Core i7-7800X, RTX 2080 GPU	AP 0.901, AP^0.5 0.910, AR^1 0.884, AR^10 0.978, AR^100 0.926	24 M Params	Not reported
[58]	Models insulators as linked chain structures.	Not reported	Smart Grid Inspection	Not reported	Not reported	Not reported
[59]	Not reported	Not reported	Not reported	Not reported	Not reported	Not reported
[60]	Not reported	CNN architecture is very complex and cannot be implemented on a computing platform with limited resources.	Computing platforms	Not reported	Not reported	Not reported
[61]	Not reported	Not reported	Not reported	Not reported	Not reported	Not reported

[62]	Multi-scale feature fusion strategy, strong generalization ability, highly balanced accuracy, and lightweight nature.	Blindly increasing model complexity is not necessarily conducive to performance improvement because large model parameters are difficult to train adequately.	UAV images, Electric power scenarios	AP50:95 57.1, AP50 86.2, AP75 64.9, AR100 65.8, APs 17.7, APm 51.3, APl 61.3	Paras 32.020 M, GFLOP 103.5	Latency 19.2 ms, FPS 52.08
[63]	Mitigates spatial misalignment using bilinear interpolation, adapts to elongated characteristics of insulators, focuses on significant features for dense small target defects.	Two-stage proposal-based method results in lower FPS; relatively high computational complexity requiring more memory and computational resources during training.	Operation and Maintenance Centers (OMCs) or Data Centers (DCs), UAV images	mAP@0.5 88.42%, Recall 81.94%	34.48 M Params, 977.53 GFLOPs	19 FPS
[64]	Real-time, high-precision defect and hazard identification using Transformer algorithm, Triplet Attention mechanism, and SIoU loss function.	Not reported	Ubuntu system, 64 GB memory, RTX 2080 Ti GPU	92.3% accuracy	Not reported	18.4 ms inference speed
[65]	Meets real-time requirement (no less than 24 FPS), achieves state-of-the-art performance on IDID dataset.	Exceeds 8 GB memory usage at image resolutions up to 4 K (4096 x 2160).	Server-side scenarios, Intel Core i7-12700 CPU, NVIDIA GeForce RTX 3060 GPU	mAP 75.8%, AP_s 20.0, AP_m 40.3, AP_l 47.8	Not reported	13.1 FPS
[66]	Synthetic fog algorithm increases fogging speed by about 40 times, realizes real-time detection on edge devices.	Small data leads to overfitting because the large model has enough	Edge devices such as Jetson nano	Not reported	311 layers	62.5 ft/s

		parameters to "remember" training data.				
[67]	Validates the efficiency of the proposed embedding matching procedure and meets the speed requirements of power line inspection.	With the increasing size, Ts shows an obvious increasing trend.	Power line inspection	Not reported	Not reported	21.6 ms
[68]	Balanced performance, lower false-positive rate and lower false-negative rate, reduces missed detections.	Not reported	Power inspections	P 93.4%, R 90.4%, mAP50 92.7%, mAP95 62.2%	2218882 Parameters, 6.6 GFLOPs, 4.6 MB Memory	38 FPS
[69]	Not reported	Not reported	Not reported	Not reported	Not reported	Not reported
[70]	Outperforms the original model across various metrics.	Not reported	Not reported	Not reported	Not reported	Not reported
[71]	Elevated detection accuracy prevents injuries, excellent generalization capabilities for broader applications.	Slower inference speed than SSD; limited distinguishability among algorithms due to small dataset.	Condition monitoring system	P 98.03%, R 95.24%, mAP@0.5 99.17%	6.68 M Paraments, 73.60 GLOPs/G	47.83 FPS
[72]	Improved average accuracy over baseline and other advanced algorithms.	Not reported	Not reported	Not reported	Not reported	0.05 sec/img
[73]	Performs best under the same criterion compared to Faster R-CNN, SSD, YOLOv5, YOLOv6, and YOLOv7.	Not reported	Not reported	P 96.8%, R 93.3%, mAP@0.5 97.1%	Not reported	Not reported
[74]	Reduces parameters and FLOPs, optimal balance between lightweighting and accuracy,	Does not demonstrate a clear advantage in terms of inference time.	Mobile platforms such as UAVs,	mAP@.5 85.16%, mAP@.5:.95 44.22%	Parameter 4.134 M, FLOPs 10.06 G	2.3 ms

	sufficient adaptability for deployment on mobile platforms.		Ubuntu 22.04, RTX 3060 GPU			
[75]	RandLA-Net is significantly more efficient than PointCNN, with a drastic reduction in inference time.	Not reported	Mobile and airborne LiDAR point clouds	Not reported	Not reported	12.1 s, 7.8 s
[76]	Not reported	Not reported	Low-Cost UAV imagery	Not reported	Not reported	Not reported
[77]	Higher accuracy and detection speed compared to R-FCN, Faster R-CNN, and YOLO.	Not reported	UAV/aerial images	Accuracy 93.5%, F_measure 92.9%, Sensitivity 90.0%, Specificity 88.4%, Balanced Accuracy 90.4%	Model size 58.2 MB	Not reported
[78]	Strikes a favorable balance between model performance and training time costs, enhanced by virtual datasets.	Not reported	Not reported	AP0.50 0.9020, AP0.75 0.8334, AP[0.50:0.95] 0.6721	Not reported	Not reported
[79]	Not reported	Not reported	PC with 3.60-GHz CPU, 32G RAM, NVIDIA GeForce RTX 2070	Not reported	Not reported	Not reported
[80]	Not reported	Not reported	Not reported	Not reported	Not reported	Not reported

[81]	Competitive solution for intelligent inspection of the power grid, provides high detection performance.	Not reported	Aerial photography/ drone	Precision 91%, Recall 98.84%, AP 100%, mAP@0.5 99.08%, F1 98%	Not reported	59 FPS
[82]	Reduces the amount of network parameters and complexity, enables running on devices with limited computing resources, integrates semantic and spatial information.	Performance decreases when ratio γ is increased to 512.	Devices with limited computing resources	Pixel Acc 99.61%, Class Acc 97.44%, mIOU 95.12%, f.w. IOU 99.24%	Parameters 13.61 M, GFLOPs 12.62	67 ms
[83]	Outperforms YOLOv5, YOLOv4, Faster R-CNN, and SSD in mAP and F1 score.	Not reported	Not reported	Precision 0.958, Recall 0.896, mAP@50 0.930, F1 0.926	16.4 GFLOPs	Not reported
[84]	Solves insulator defect detection under extreme weather, improves generalization capability for insulators and defects.	Not reported	Real-time drone power inspection	P 93.4%, R 86.5%, mAP 91.3%	Not reported	46.5 FPS
[85]	Not reported	Not reported	Not reported	Not reported	Not reported	Not reported
[86]	Overcomes environmental challenges, lightweight architecture, minimal parameter cost, adaptive robustness module mitigates noise.	Not reported	UAVs (Raspberry Pi 4, Intel Realsense T265 camera), edge computing devices	P 93.9%, R 94.2%, F2-score 94.0, mAP@0.5 97.7%, mAP@0.5:0.95 80.4%	1.61m parameters, 3.9 GFLOPs, 3.38m weights	10.5 ms, 40 FPS

[87]	Achieves significant performance gains, good image restoration, parameter efficient and well-suited for deployment in engineering applications.	Not reported	Engineering applications	mAP@50 89.6%, precision 93.0%, mAP@50:95 65.9%	2.58 M parameters	Not reported
[88]	Parameter-efficient, high detection accuracy without much enhancement in computation time, attains optimal balance between detection accuracy and computational efficiency.	Slightly higher computational complexity, inference time, parameters, memory footprint size, and GFLOPs compared to the baseline YOLOv7.	UAV images	mAP@50 90.30%, Precision 86.9%, Recall 87.7%, F1 87.3%	38.2 M Parameters, 108.7 GFLOPs, 78.4 MB model size	12.3 ms
[89]	Lightweight power equipment detection, significantly reduces model parameters, highly suitable for deployment on resource-constrained edge devices.	Not reported	Edge devices	Precision 88.1%, Recall 87.5%, mAP 90.0%	Params 5.0 M, GFLOPs 11.9, Size 9.8 MB	137.1 FPS (GPU), 12.7 FPS (CPU)
[90]	Multi-guess capability, ordered loss, and ConvNeXt improve F1 and Fbeta scores; good capability in weakly supervised settings.	Increased capability comes at the cost of efficiency (higher inference time, more parameters than HAWPv2).	Tested on RTX 3090, needs future work to support real-time applications	APR 0.845, ARR 0.814, F1 0.829, Fbeta 0.837	13.9 million parameters, 41.9 GFLOPs	0.17 s
[91]	Not reported	Not reported	Not reported	Not reported	Not reported	Not reported
[92]	High performance, broader applicability via cross-domain evaluation, extremely lightweight, enables real-time operation on embedded or edge devices, identifies missed defects.	Inference speed is slightly lower than YOLOv8n's (150.6 FPS).	Embedded or edge devices, 12-core Intel Xeon CPU, GPU	mAP@0.5 94.2%	1.3 M parameters, 4.4 G FLOPs, 3.1 MB model size	123.8 FPS

[93]	Branchless structure and efficient ReLU reduce memory accesses and improve FLOPS, suitable for deployment in resource-limited edge computing devices.	Not reported	Resource-limited edge computing devices (NVIDIA Jetson NX)	mAP 67.48%	18.95 GFLOPs	31.1 FPS
[94]	Validates the detection performance using binary precision and recall.	Not reported	NVIDIA GTX 1080Ti GPU on Ubuntu 16.04	Not reported	Not reported	Not reported
[95]	Automatic processing in a fraction of time compared to manual inspection, faster and more efficient single-stage methods.	Erroneous and missed detections (e.g., steel incorrectly identified as defect, moss identified as crack).	Not reported	Not reported	Not reported	Not reported
[96]	Improved average accuracy of the model significantly.	Not reported	i9-10900K@3.7 GHz, NVIDIA 2*RTX3090 24 GB	mAP 94.7%	Not reported	0.05 sec/img
[97]	Not reported	Not reported	Not reported	Not reported	Not reported	Not reported
[98]	High recognition speed and accuracy, streamlines dataset to improve generalization and efficiency.	AdaBoost's inference time lengthens with the number of estimators; ResNet-50_XgBoost performs poorly.	UAV images	Training accuracy 91.874%, AUC 0.9856	253953 parameter count	430 s, 152 s
[99]	Superior parameter utilization efficiency and practical balance; multi-scale feature fusion and attention mechanism have strong	Not reported	UAV-based object detection tasks, NVIDIA RTX	AP50 0.910, AP75 0.763, AP50–95 0.662	21.123 M parameters, 59.4 GFLOPs	190 FPS

	generalization ability and robustness.		4090 GPU (desktop-class hardware)			
[100]	Reduces computational costs by estimating the class prior efficiently without multiple training processes.	Not reported	Not reported	77.03% AP, 88.72% AP@0.75, 93.74% AP@0.5	Not reported	Not reported
[101]	Not reported	Not reported	Not reported	Not reported	Not reported	Not reported
[102]	Effortless approach, consistent results, ability to reach remote areas conveniently.	Not reported	UAV, aircrafts operating at low altitudes	AP 56.5 ± 1.06%, AP_OKS=.50 88.2 ± 1.13%, AR 68.2 ± 0.91%, F1-Score 61.70 ± 0.91%	3.36 M parameters	42.71 FPS
[103]	Not reported	Not reported	Not reported	Not reported	Not reported	Not reported
[104]	Superior performance compared to Cutout, operates at the feature level.	Not reported	Not reported	Not reported	Not reported	Not reported
[105]	Makes full use of the line features and location information	Difficult to maintain high accuracy or generalization ability in scenes with complex backgrounds.	Aerial images	TPR 0.9941, FPR 0.0053	Not reported	0.27 s processing time
[106]	Better fits the distribution and expression information of power line images.	Results may deviate if obscured by large areas; modeling process is slightly complicated.	Aerial images	Not reported	Not reported	Not reported
[107]	Higher precision means fewer false positives.	Not reported	Airborne LiDAR	Not reported	Not reported	Not reported

[108]	Steady performance improvement across evaluation metrics, training convergence.	Not reported	Not reported	Not reported	Not reported	Not reported
[109]	Autonomous perception and motion planning, binocular image obtaining.	Not reported	UAV (DJI M300) with NVIDIA Jetson AGX Xavier	Not reported	Not reported	Not reported
[110]	Automatically extracts features, strong generalizability and resilience against noise.	Not fully validated across various designs; potential overfitting; latency/memory issues in real time.	Aerial Surveillance (UAV)	Not reported	Not reported	Not reported
[111]	Improves detection accuracy, reduces computational complexity, enhances robustness.	Mutual exclusivity between Precision and Recall based on IoU threshold.	UAVs equipped with edge computing devices	AP 65.7%, AP50 88.4%, AP75 76.2%	2.79 Million Parameters, Size 5.9 MB	Not reported
[112]	Not reported	Not reported	UAV	Not reported	Not reported	Not reported
[113]	Real-time suitability, acceptable frame rate for real-time processing.	Accuracy dropped after weight optimization for YOLOv4-tiny.	UAVs, Edge Computing (Nvidia Jetson AGX Xavier)	Accuracy 84.4%, loss 0.15	Not reported	22 FPS
[114]	Effective and less risky; real-time processing on lightweight edge devices.	Some optimization techniques required for Raspberry Pi 4; limited benefits from data augmentation.	Edge Computing (Raspberry Pi 4B, Jetson Nano, Jetson Orin Nano), Drones	mAP 90.80%, F1-score 90.90%, Pr 89.80%, Rc 89.42%	Not reported	Not reported

[115]	Consistent measurement accuracy, faster and more automated for image processing.	Not reported	Overhead Transmission Line	Not reported	Not reported	Not reported
[116]	Enhances perception/generalization; effectively captures shape information.	Not reported	Edge devices like UAVs	mAP50 99.01%, Precision 99.42%, Recall 95.13%	45.835 Param, 117.1 GFLOPs	Not reported
[117]	Reduces model size; meets practical engineering requirements for flexibility/reliability.	Not reported	Embedded hardware devices, drones	Not reported	Not reported	Not reported
[118]	Enhances global context modeling with linear cost; strong adaptability to fog/blur/terrain.	Sensitive to optical degradation; error sources come from loss of detail in icy edges.	Edge devices like UAVs, drone/pole terminal	mAP50 87.1%, Precision 90.0%, Recall 95.3%	12.5 M parameters, 3.2 G FLOPs	66 FPS, 15.2 ms latency
[119]	Not reported	Not reported	Not reported	Not reported	Not reported	Not reported
[120]	Not reported	Decrease in speed by 5.5 images per second compared to Faster R-CNN.	UAV images	Not reported	Not reported	Not reported
[121]	Not reported	Not reported	Not reported	Not reported	Not reported	Not reported
[122]	Not reported	F1 of missing vibration damper detection is low; tower detection performance is insufficient.	UAV-taken images	Not reported	Not reported	Not reported
[123]	Alleviates problems caused by hard/easy sample imbalance.	Not reported	Not reported	Not reported	Not reported	Not reported

[124]	Not reported	Not reported	Not reported	Not reported	Not reported	Not reported
[125]	Markedly reduced level of complexity in comparison to alternative algorithms.	Not reported	Not reported	AP 0.937, mAP0.5 0.912, mAP0.5-0.95 0.737	2.82 M Parameters, 7.6 GFLOPs	208 FPS
[126]	Not reported	Not reported	Not reported	Not reported	Not reported	Not reported
[127]	Significant boost in accuracy without a substantial increase in parameters.	Limited generalizability across different foreign body types; struggles with background texture.	Hardware with limited resources	P 97.8%, R 92.5%, mAP 97.6%	3,249,024 Parameters	269.41 FPS
[128]	Unaffected by external light conditions, offers accurate measurements and convenience.	Not reported	UAV equipped with IR cameras	mAP 97.40%	Model size 135 MB	33.4 FPS
[129]	Semantic target guides representation learning without additional memory	Unacceptable performance when trained from scratch without pretraining.	Not reported	Not reported	Not reported	Not reported
[130]	Enables preliminary on-board detection and filtering to reduce downstream bandwidth/storage.	Not reported	UAV (DJI Matrice 300 RTK), NVIDIA Jetson Xavier NX	Not reported	Not reported	Not reported
[131]	Not reported	Not reported	UAV Patrol Images	Not reported	Not reported	Not reported
[132]	Outperforms cutting-edge techniques while balancing high performance with low complexity.	Not reported	Mobile devices, resource-constrained drones	Not reported	16.86 M parameters, 6.41 G FLOPs	Not reported

[133]	Not reported	Traditional mathematical models require intense calculation and human resources.	Not reported	Not reported	Not reported	Not reported
[134]	Not reported	Not reported	Not reported	mAP 0.948	426.53 Flops	Not reported
[135]	Reduces inference delay by 12 ms via better channel-attention mechanisms.	Not reported	Edge-Side Deployment (Jetson Xavier NX on UAV)	mAP 93.5%	3.1 MB Params, 4.3 G FLOPs	23.5 ± 2.2 FPS
[136]	Not reported	Not reported	UAV, Edge-computing	Not reported	Not reported	Not reported
[137]	Not reported	Complex architecture increases parameter count; limits embedded inference speed.	Desktop GPU (NVIDIA GTX 4090)	Not reported	Not reported	Not reported
[138]	High precision, high automation, short mapping periods.	Complex scenarios (intersecting lines, proximity to trees) degrade evaluation metrics.	Mobile LiDAR	Precision 0.95, Recall 0.79, F1score 0.86	Not reported	Not reported
[139]	Higher spatial resolution than satellite; cheaper and enables near real-time segmentation.	Labeling is challenging due to massive areas and high resolution.	UAV optical images	Not reported	Not reported	Not reported
[140]	Not reported	Not reported	Not reported	Not reported	Not reported	Not reported
[141]	Competitive performance on power line detection.	Shortcomings still exist in achieving extremely high-precision boundary detection.	Not reported	Not reported	Not reported	Processing time 0.051 s
[142]	Not reported	Not reported	Not reported	Not reported	Not reported	Not reported

[143]	Not reported	Not reported	UAV	mAP50 46.72%, mAP95 34.38%, P 21.4%, R 15.8%	7.2 M Params, 21.4 G FLOPs	Not reported
[144]	Not reported	Not reported	NVIDIA A40 GPU	Not reported	Not reported	Not reported
[145]	Not reported	Not reported	Not reported	Not reported	Not reported	Not reported
[146]	Not reported	Direct video input/real-time drone applications require further engineering constraints.	Drone-based system (pre-recorded processing currently)	Not reported	Not reported	Not reported
[147]	Balances precision and computation effectively for edge GPUs.	Normalization perturbation shows limited effectiveness under uniform noise, such as fog.	UAV edge hardware (Jetson Nano/Orin)	Not reported	Not reported	~100 FPS
[148]	Significant advantage in speed-accuracy trade-off; good generalization performance.	Not reported	Server (GeForce RTX 3090) / Autonomous Aerial Vehicle cloud platform	Not reported	Not reported	Not reported

References

1. Hao, S.; Li, T.; Ma, X.; Fan, S.; Qi, T. A Decoupled Hierarchical Fault Detection Method for Insulator Parallel Gaps: Integrating Lightweight Localization and Attention-Based Diagnosis. *IEEE Trans. Instrum. Meas.* **2025**, *74*, 1–15, doi:10.1109/TIM.2025.3604115.
2. Ji, T.; Liu, S. A Directional Defect Detector for Corona Ring Fault Diagnosis in Power Transmission Line. *IEEE Trans. Instrum. Meas.* **2025**, *74*, 1–14, doi:10.1109/TIM.2025.3569353.

3. Siddiqui, Z.A.; Park, U. A Drone Based Transmission Line Components Inspection System with Deep Learning Technique. *Energies* **2020**, *13*, 3348, doi:10.3390/en13133348.

4. Zhang, J.; Lei, J.; Qin, X.; Li, B.; Li, Z.; Li, H.; Zeng, Y.; Song, J. A Fitting Recognition Approach Combining Depth-Attention YOLOv5 and Prior Synthetic Dataset. *Appl. Sci.* **2022**, *12*, 11122, doi:10.3390/app122111122.

5. Lu, Y.; Li, D.; Li, D.; Li, X.; Gao, Q.; Yu, X. A Lightweight Insulator Defect Detection Model Based on Drone Images. *Drones* **2024**, *8*, 431, doi:10.3390/drones8090431.

6. Wang, Y.; Zhang, L.; Xiong, X.; Kuang, J.; Xiang, S. A Lightweight and Efficient Multi-Type Defect Detection Method for Transmission Lines Based on DCP-YOLOv8. *Sensors* **2024**, *24*, 4491, doi:10.3390/s24144491.

7. Jia, Z.; Ouyang, Y.; Feng, C.; Fan, S.; Liu, Z.; Sun, C. A Live Detecting System for Strain Clamps of Transmission Lines Based on Dual UAVs' Cooperation. *Drones* **2024**, *8*, 333, doi:10.3390/drones8070333.

8. Han, J.; Yang, Z.; Zhang, Q.; Chen, C.; Li, H.; Lai, S.; Hu, G.; Xu, C.; Xu, H.; Wang, D.; et al. A Method of Insulator Faults Detection in Aerial Images for High-Voltage Transmission Lines Inspection. *Appl. Sci.* **2019**, *9*, 2009, doi:10.3390/app9102009.

9. Zhao, Z.; Wang, R.; Li, Y.; Zhai, Y.; Zhao, W.; Zhang, K. A New Multilabel Recognition Framework for Transmission Lines Bolt Defects Based on the Combination of Semantic Knowledge and Structural Knowledge. *IEEE Trans. Instrum. Meas.* **2022**, *71*, 1–11, doi:10.1109/TIM.2022.3200103.

10. Yan, J.; Zhang, X.; Shen, S.; He, X.; Xia, X.; Li, N.; Wang, S.; Yang, Y.; Ding, N. A Real-Time Strand Breakage Detection Method for Power Line Inspection with UAVs. *Drones* **2023**, *7*, 574, doi:10.3390/drones7090574.

11. Huo, Y.; Dai, X.; Tang, Z.; Xiao, Y.; Zhang, Y.; Fang, X. A Three-Granularity Pose Estimation Framework for Multi-Type High-Voltage Transmission Towers Using Part Affinity Fields (PAFs). *Energies* **2025**, *18*, 488, doi:10.3390/en18030488.

12. Jain, N.; Bedi, J.; Anand, A.; Godara, S. A Transfer Learning Architecture to Detect Faulty Insulators in Powerlines. *IEEE Trans. Power Deliv.* **2024**, *39*, 1002–1011, doi:10.1109/TPWRD.2024.3353203.

13. He, M.; Qin, L.; Wang, Y.; Deng, X.; Liu, Q.; Zhang, Y.; Liu, K. A Weakly Supervised Contrastive Learning Pretraining Method for Visual Defect Detection of Transmission Lines. *IEEE Trans. Instrum. Meas.* **2025**, *74*, 1–15, doi:10.1109/TIM.2025.3577837.

14. Yang, L.; Fan, J.; Song, S.; Liu, Y. A Light Defect Detection Algorithm of Power Insulators from Aerial Images for Power Inspection. *Neural Comput. Appl.* **2022**, *34*, 17951–17961, doi:10.1007/s00521-022-07437-5.

15. Wu, J.; Wang, W.; Du, W.; Liu, Z.; Lin, L.; Cui, Y.; Wang, C.; Tian, L. A Lightweight Insulator Defect Detection Algorithm Based on Drone Images for Power Line Inspection. *Eng. Res. Express* **2025**, *7*, 45355, doi:10.1088/2631-8695/ae19ce.

16. Xu, M.; Zhong, X.; Huang, J.; Ma, H.; Zhong, R. A Method for Accurately Extracting Power Lines and Identifying Potential Intrusion Risks from Urban Laser Scanning Data. *Opt. Lasers Eng.* **2024**, *174*, 107987, doi:10.1016/j.optlaseng.2023.107987.

17. Zhao, W.; Dong, Q.; Zuo, Z. A Point Cloud Segmentation Method for Power Lines and Towers Based on a Combination of Multi-scale Density Features and Point-Based Deep Learning. *Int. J. Digit. Earth* **2023**, *16*, 620–644, doi:10.1080/17538947.2023.2168770.

18. Li, W.; Tong, Q.; Gu, J.; Li, J.; Sun, W.; Li, Q. A Self-Adjusting Transformer Network for Detecting Transmission Line Defects. *Neural Comput. Appl.* **2024**, *36*, 4467–4484, doi:10.1007/s00521-023-09319-w.

19. Sim, Y.-S.; Lee, C.-K.; Hwang, J.-S.; Kwon, G.-Y.; Chang, S.J. AI-Based Remaining Useful Life Prediction for Transmission Systems: Integrating Operating Conditions with TimeGAN and CNN-LSTM Networks. *Electr. Power Syst. Res.* **2025**, *238*, 111151, doi:10.1016/j.epsr.2024.111151.

20. Lan, K.; Zheng, X.; Li, S.; Hu, J.; Shao, L. AMFA-Net: Aerial Power Line Segmentation Network Based on Attention Mechanism and Feature Aggregation. *Measurement* **2026**, *257*, 118684, doi:10.1016/j.measurement.2025.118684.

21. Zhang, T.; Tan, J.; Li, S.; Yang, R. APE: Anomaly-Guided Progressively Balanced Ensemble Learning for Insulator Extraction From Imbalanced LiDAR Data. *IEEE Trans. Geosci. Remote Sens.* **2025**, *63*, 1–19, doi:10.1109/TGRS.2025.3574343.

22. Sankuri, R.S.; Sristy, N.B.; Karri, S.P.K. Accurate Insulator Defect Detection in Power Transmission Lines Using Semi-Supervised Hybrid DETR with Advanced Loss Methods. *J. Real-Time Image Process.* **2025**, *22*, 181, doi:10.1007/s11554-025-01760-5.

23. Cheng, Y.; Liu, D. AdIn-DETR: Adapting Detection Transformer for End-to-End Real-Time Power Line Insulator Defect Detection. *IEEE Trans. Instrum. Meas.* **2024**, *73*, 1–11, doi:10.1109/TIM.2024.3420265.

24. Bellou, E.; Pisica, I.; Banitsas, K. Aerial Inspection of High-Voltage Power Lines Using YOLOv8 Real-Time Object Detector. *Energies* **2024**, *17*, 2535, doi:10.3390/en17112535.

25. Dai, G.; Wang, C.; Tang, Q.; He, L.; Chen, H.; Sunil, C.K.; Dewi, C. Aerial Infrared Thermal Imaging Transmission Line Defect Detection Methods Incorporating Explicit Visual Center Structures. *Measurement* **2025**, *245*, 116605, doi:10.1016/j.measurement.2024.116605.

26. Wen, C.; Li, X.; Yao, X.; Peng, L.; Chi, T. Airborne LiDAR Point Cloud Classification with Global-Local Graph Attention Convolution Neural Network. *ISPRS J. Photogramm. Remote Sens.* **2021**, *173*, 181–194, doi:10.1016/j.isprsjprs.2021.01.007.

27. Zhang, J.; Wang, J.; Song, R.; Peng, G.; Pu, T.; Zhang, S. An Edge Visual Incremental Perception Framework Based on Deep Semi-Supervised Learning for Monitoring Power Transmission Lines. *CSEE J. Power Energy Syst.* **2023**, *9*, 759–768, doi:10.17775/CSEEJPES.2022.03120.

28. Nusantika, N.R.; Xiao, J.; Hu, X. An Enhanced Multi-scale Retinex, Oriented FAST and Rotated BRIEF (ORB), and Scale-Invariant Feature Transform (SIFT) Pipeline for Robust Key Point Matching in 3D Monitoring of Power Transmission Line Icing with Binocular Vision. *Electronics* **2024**, *13*, 4252, doi:10.3390/electronics13214252.

29. Zhang, X.; Zhang, J.; Jia, X. An Enhanced SL-YOLOv8-Based Lightweight Remote Sensing Detection Algorithm for Identifying Broken Strands in Transmission Lines. *Appl. Sci.* **2024**, *14*, 7469, doi:10.3390/app14177469.

30. Dongfeng, P.; Wei, G.; Ming, L.; Xuwu, G.; Zhengkun, Z.; Shuohe, W. An Improved Lightweight RT-DETR Algorithm for High-Voltage Transmission Line Inspection. *IEEE Access* **2025**, *13*, 183408–183418, doi:10.1109/ACCESS.2025.3625122.

31. Zhao, W.; Xu, M.; Cheng, X.; Zhao, Z. An Insulator in Transmission Lines Recognition and Fault Detection Model Based on Improved Faster RCNN. *IEEE Trans. Instrum. Meas.* **2021**, *70*, 1–8, doi:10.1109/TIM.2021.3112227.

32. Zhang, J.; Wang, J.; Zhang, S. An Ultra-Lightweight and Ultra-Fast Abnormal Target Identification Network for Transmission Line. *IEEE Sens. J.* **2021**, *21*, 23325–23334, doi:10.1109/JSEN.2021.3110878.

33. Zhang, J.; Wang, X.; Li, Y.; Li, D.; Wang, F.; Li, L.; Zhang, H.; Shi, X. An Adaptive Dual-Weighted Feature Network for Insulator Detection in Transmission Lines. *Neural Comput. Appl.* **2025**, *37*, 7067–7087, doi:10.1007/s00521-024-10957-x.

34. Yu, X.; Zhou, Z.; Deng, Y.; Zhang, K.; Gu, C.; Liu, Z.; Zhou, S. An Angle-Enhanced Deep Learning Framework for Thermal Defect Diagnosis in Overhead Transmission Line Composite Insulators. *Eng. Appl. Artif. Intell.* **2025**, *162*, 112285, doi:10.1016/j.engappai.2025.112285.

35. You, X.; Ma, J.; Yang, G. An Improved Insulator Self-Explosion Detection Method Based on Group-Level Pruning for the YOLOv7-Tiny Algorithm. *J. Real-Time Image Process.* **2024**, *21*, 189, doi:10.1007/s11554-024-01571-0.

36. Wu, T.; Wang, L.; Xu, X.; Su, L.; He, W.; Wang, X. An Intelligent Fault Detection Algorithm for Power Transmission Lines Based on Multi-Scale Fusion. *Intell. Robot.* **2025**, *5*, 474–487, doi:10.20517/ir.2025.24.

37. Wang, J.; Jin, L.; Li, Y.; Cao, P. Application of End-to-End Perception Framework Based on Boosted DETR in UAV Inspection of Overhead Transmission Lines. *Drones* **2024**, *8*, 545, doi:10.3390/drones8100545.

38. Liu, C.; Liu, J.; Wu, Y.; Sun, Z. Application of Enhanced YOLOv8 in Multi-Object Detection for Autonomous Inspection of Transmission Lines. *Eng. Res. Express* **2025**, *7*, 45240, doi:10.1088/2631-8695/ae15e6.

39. Choi, H.; Yun, J.P.; Kim, B.J.; Jang, H.; Kim, S.W. Attention-Based Multimodal Image Feature Fusion Module for Transmission Line Detection. *IEEE Trans. Ind. Informatics* **2022**, *18*, 7686–7695, doi:10.1109/TII.2022.3147833.

40. Zhang, H.; Wu, L.; Chen, Y.; Chen, R.; Kong, S.; Wang, Y.; Hu, J.; Wu, J. Attention-Guided Multitask Convolutional Neural Network for Power Line Parts Detection. *IEEE Trans. Instrum. Meas.* **2022**, *71*, 1–13, doi:10.1109/TIM.2022.3162615.

41. Jung, J.; Che, E.; Olsen, M.J.; Shafer, K.C. Automated and Efficient Powerline Extraction from Laser Scanning Data Using a Voxel-Based Subsampling with Hierarchical Approach. *ISPRS J. Photogramm. Remote Sens.* **2020**, *163*, 343–361, doi:10.1016/j.isprsjprs.2020.03.018.

42. Li, D.; Wang, X.; Zhang, J.; Ji, Z. Automated Deep Learning System for Power Line Inspection Image Analysis and Processing: Architecture and Design Issues. *Glob. Energy Interconnect.* **2023**, *6*, 614–633, doi:10.1016/j.gloei.2023.10.008.

43. Wang, M. Automated Monitoring of High-Voltage Transmission Lines Based on Machine Vision. *Appl. Math. Nonlinear Sci.* **2024**, *9*, doi:10.2478/amns-2024-0077.

44. Xiang, Y.; Du, C.; Mei, Y.; Zhang, L.; Du, Y.; Liu, A. BN-YOLO: A Lightweight Method for Bird's Nest Detection on Transmission Lines. *J. Real-Time Image Process.* **2024**, *21*, 194, doi:10.1007/s11554-024-01577-8.

45. Li, Y.; Qian, Q.; Duan, H.; Min, X.; Xu, Y.; Jiang, X. Boosting Power Line Inspection in Bad Weather: Removing Weather Noise with Channel-Spatial Attention-Based UNet. *Multimed. Tools Appl.* **2023**, *83*, 88429–88445, doi:10.1007/s11042-023-17554-5.

46. Liu, X.; Miao, X.; Jiang, H.; Chen, J. Box-Point Detector: A Diagnosis Method for Insulator Faults in Power Lines Using Aerial Images and Convolutional Neural Networks. *IEEE Trans. Power Deliv.* **2021**, *36*, 3765–3773, doi:10.1109/TPWRD.2020.3048935.

47. Dai, Z.; Yi, J.; Jiang, L.; Yang, S.; Huang, X. Cascade CenterNet: Robust Object Detection for Power Line Surveillance. *IEEE Access* **2021**, *9*, 60244–60257, doi:10.1109/ACCESS.2021.3072901.

48. Xu, Q.; Zhang, Y.; Peng, H.; Liu, Y.; Xu, D.; Cai, S.; Li, P. Cloud-Edge-End Collaboration for Power Line Inspection: A Proximal Policy Optimisation-Based Image Offloading Approach to Minimize Task Execution Time. *Eng. Res. Express* **2025**, *7*, 45313, doi:10.1088/2631-8695/ae0de6.

49. Zhao, L.; Liu, C.; Qu, H. Co-Occurrence Object Detection of the Transmission Lines Based on the Cross-Domain Interactive Feature Enhancement. *IEEE Trans. Power Deliv.* **2023**, *38*, 4443–4453, doi:10.1109/TPWRD.2023.3321867.

50. Wu, J.; Sun, Y.; Wang, X. Corrosion Detection Method of Transmission Line Components in Mining Area Based on Multi-scale Enhanced Fusion. *Mob. Inf. Syst.* **2022**, *2022*, 1–12, doi:10.1155/2022/7408265.

51. Wu, D.; Yang, W.; Li, J.; Du, K.; Li, L.; Yang, Z. CRL-YOLO: A Comprehensive Recalibration and Lightweight Detection Model for AAV Power Line Inspections. *IEEE Trans. Instrum. Meas.* **2025**, *74*, 1–21, doi:10.1109/TIM.2025.3562980.

52. Wang, Y.; Qu, Z.; Hu, Z.; Yang, C.; Huang, X.; Zhao, Z.; Zhai, Y. Cross-Domain Multilevel Feature Adaptive Alignment R-CNN for Insulator Defect Detection in Transmission Lines. *IEEE Trans. Instrum. Meas.* **2025**, *74*, 1–12, doi:10.1109/TIM.2025.3527619.

53. Hao, S.; Li, G.-L.; Ma, X.; Qi, T.; Li, T.; Fan, S. DM-YOLO: Transmission Line Fault Detection Based on Dynamic Multi-Scale Convolution and Attention Mechanism. *Meas. Sci. Technol.* **2025**, *36*, 106109, doi:10.1088/1361-6501/ae08d7.

54. Yang, L.; Kong, S.; Deng, J.; Li, H.; Liu, Y. DRA-Net: A Dual-Branch Residual Attention Network for Pixelwise Power Line Detection. *IEEE Trans. Instrum. Meas.* **2023**, *72*, 1–13, doi:10.1109/TIM.2023.3259047.

55. Zhang, Y.; Li, B.; Shang, J.; Huang, X.; Zhai, P.; Geng, C. DSA-Net: An Attention-Guided Network for Real-Time Defect Detection of Transmission Line Dampers Applied to UAV Inspections. *IEEE Trans. Instrum. Meas.* **2024**, *73*, 1–22, doi:10.1109/TIM.2023.3331418.

56. Maduako, I.; Igwe, C.F.; Abah, J.E.; Onwuasaanya, O.E.; Chukwu, G.A.; Ezeji, F.; Okeke, F.I. Deep Learning for Component Fault Detection in Electricity Transmission Lines. *J. Big Data* **2022**, *9*, 81, doi:10.1186/s40537-022-00630-2.

57. Gong, Y.; Zhou, W.; Wang, K.; Wang, J.; Wang, R.; Deng, H.; Liu, G. Defect Detection of Small Cotter Pins in Electric Power Transmission System from UAV Images Using Deep Learning Techniques. *Electr. Eng.* **2023**, *105*, 1251–1266, doi:10.1007/s00202-022-01729-8.

58. Wei, N.; Li, X.; Jin, J.; Chen, P.; Sun, S. Detecting Insulator Strings as Linked Chain Structure in Smart Grid Inspection. *IEEE Trans. Ind. Informatics* **2023**, *19*, 9019–9027, doi:10.1109/TII.2022.3224956.

59. Liang, H.; Zuo, C.; Wei, W. Detection and Evaluation Method of Transmission Line Defects Based on Deep Learning. *IEEE Access* **2020**, *8*, 38448–38458, doi:10.1109/ACCESS.2020.2974798.

60. Zhang, Z.; Chen, H.; Huang, S. Detection of Missing Insulator Caps Based on Machine Learning and Morphological Detection. *Sensors* **2023**, *23*, 1557, doi:10.3390/s23031557.

61. Zhang, Y.; Dou, Y.; Zhao, L.; Jiao, Y.; Guo, D. EDUNet++: An Enhanced Denoising Unet++ for Ice-Covered Transmission Line Images. *Electronics* **2024**, *13*, 2085, doi:10.3390/electronics13112085.

62. Yang, K.; Zhai, B.; Zhao, X.; Wang, Q.; Zhai, Y. EPFD: An Electric Power Fitting Dataset and Benchmark for Object Detection. *IEEE Trans. Ind. Informatics* **2025**, *21*, 9183–9193, doi:10.1109/TII.2025.3598424.

63. Wang, Q.; Li, Y.; Cui, S.; Li, N.; Zhang, X.; Jiang, W.; Peng, W.; Sun, J. Enhanced Recognition of Insulator Defects on Power Transmission Lines via Proposal-Based Detection Model with Integrated Improvement Methods. *Eng. Appl. Artif. Intell.* **2024**, *136*, 109078, doi:10.1016/j.engappai.2024.109078.

64. Wang, M.; Sun, L.; Jiang, J.; Yang, J.; Zhang, X. Enhancing Hyperspectral Power Transmission Line Defect and Hazard Identification with an Improved YOLO-Based Model. *Optoelectron. Lett.* **2024**, *20*, 681–688, doi:10.1007/s11801-024-3213-3.

65. Zhang, X.; Zhang, Y.; Shen, K.; Fu, Q.; Shen, H. FAFNet: An Overhead Transmission Line Component Detection Method Based on Feature Alignment and Fusion. *IEEE Sens. J.* **2025**, *25*, 30197–30206, doi:10.1109/JSEN.2025.3581017.

66. Zhang, Z.-D.; Zhang, B.; Lan, Z.-C.; Liu, H.-C.; Li, D.-Y.; Pei, L.; Yu, W.-X. FINet: An Insulator Dataset and Detection Benchmark Based on Synthetic Fog and Improved YOLOv5. *IEEE Trans. Instrum. Meas.* **2022**, *71*, 1–8, doi:10.1109/TIM.2022.3194909.

67. Liu, X.; Miao, X.; Jiang, H.; Chen, J.; Chen, Z. Fault Diagnosis in Power Line Inspection Using Normalized Multihierarchy Embedding Matching. *IEEE Trans. Instrum. Meas.* **2023**, *72*, 1–10, doi:10.1109/TIM.2023.3238696.

68. Wu, D.; Yang, W.; Li, J. Fault Detection Method for Transmission Line Components Based on Lightweight GMPPD-YOLO. *Meas. Sci. Technol.* **2024**, *35*, 116015, doi:10.1088/1361-6501/ad7310.

69. Yu, C.; Liu, Y.; Zhang, W.; Zhang, X.; Zhang, Y.; Jiang, X. Foreign Objects Identification of Transmission Line Based on Improved YOLOv7. *IEEE Access* **2023**, *11*, 51997–52008, doi:10.1109/ACCESS.2023.3277954.

70. Wang, Z.; Yuan, G.; Zhou, H.; Ma, Y.; Ma, Y. Foreign-Object Detection in High-Voltage Transmission Line Based on Improved YOLOv8m. *Appl. Sci.* **2023**, *13*, 12775, doi:10.3390/app132312775.

71. Ji, C.; Jia, X.; Huang, X.; Zhou, S.; Chen, G.; Zhu, Y. FusionNet: Detection of Foreign Objects in Transmission Lines During Inclement Weather. *IEEE Trans. Instrum. Meas.* **2024**, *73*, 1–18, doi:10.1109/TIM.2024.3403173.

72. Zhai, Y.; Chen, N.; Guo, C.; Wang, Q.; Wang, Y. Graph Convolution Detection Method of Transmission Line Fitting Based on Orientation Reasoning. *Signal, Image Video Process.* **2024**, *18*, 3603–3614, doi:10.1007/s11760-024-03025-3.

73. Wang, S.; Tan, W.; Yang, T.; Zeng, L.; Hou, W.; Zhou, Q. High-Voltage Transmission Line Foreign Object and Power Component Defect Detection Based on Improved YOLOv5. *J. Electr. Eng. Technol.* **2024**, *19*, 851–866, doi:10.1007/s42835-023-01625-6.

74. Lirong, L.; Hao, C.; Junwei, D.; Bing, M.; Zhijie, Z.; Zhangqin, H.; Jie, L. High-Voltage Transmission Line Inspection Based on Multi-Scale Lightweight Convolution. *Measurement* **2025**, *251*, 117193, doi:10.1016/j.measurement.2025.117193.

75. Al-Najjar, A.; Amini, M.; Rajan, S.; Green, J.R. Identifying Areas of High-Risk Vegetation Encroachment on Electrical Powerlines Using Mobile and Airborne Laser Scanned Point Clouds. *IEEE Sens. J.* **2024**, *24*, 22129–22143, doi:10.1109/JSEN.2023.3348785.

76. Fryskowska, A. Improvement of 3D Power Line Extraction from Multiple Low-Cost UAV Imagery Using Wavelet Analysis. *Sensors* **2019**, *19*, 700, doi:10.3390/s19030700.

77. Ahmed, M.F.; Mohanta, J.C.; Sanyal, A. Inspection and Identification of Transmission Line Insulator Breakdown Based on Deep Learning Using Aerial Images. *Electr. Power Syst. Res.* **2022**, *211*, 108199, doi:10.1016/j.epsr.2022.108199.

78. Chen, Z.; Ji, Y.; Du, X.; Zhao, S.; Huo, Z.; Fang, X. Instance Segmentation Method for Insulators in Complex Backgrounds Based on Improved SOLOv2. *Sensors* **2025**, *25*, 5318, doi:10.3390/s25175318.

79. Zhang, X.; Zhang, Y.; Liu, J.; Zhang, C.; Xue, X.; Zhang, H.; Zhang, W. InsuDet: A Fault Detection Method for Insulators of Overhead Transmission Lines Using Convolutional Neural Networks. *IEEE Trans. Instrum. Meas.* **2021**, *70*, 1–12, doi:10.1109/TIM.2021.3120796.

80. Wang, Z.; Gao, Q.; Li, D.; Liu, J.; Wang, H.; Yu, X.; Wang, Y. Insulator Anomaly Detection Method Based on Few-Shot Learning. *IEEE Access* **2021**, *9*, 94970–94980, doi:10.1109/ACCESS.2021.3071305.

81. Wang, S.; Zou, X.; Zhu, W.; Zeng, L. Insulator Defects Detection for Aerial Photography of the Power Grid Using You Only Look Once Algorithm. *J. Electr. Eng. Technol.* **2023**, *18*, 3287–3300, doi:10.1007/s42835-023-01385-3.

82. Cui, Z.; Yang, C.; Wang, S. Insulator Semantic Segmentation in Aerial Images Based on Multi-scale Feature Fusion. *Complexity* **2022**, *2022*, doi:10.1155/2022/2468431.

83. Zhang, Y.; Dou, Y.; Yang, K.; Song, X.; Wang, J.; Zhao, L. Insulator Defect Detection Based on BaS-YOLOv5. *Multimed. Syst.* **2024**, *30*, 212, doi:10.1007/s00530-024-01413-w.

84. Yang, Y.; Yang, S.; Li, C.; Wang, Y.; Pi, X.; Lu, Y.; Wu, R. Insulator Defect Detection under Extreme Weather Based on Synthetic Weather Algorithm and Improved YOLOv7. *High Volt.* **2025**, *10*, 69–77, doi:10.1049/hve2.12513.

85. Wang, F.; Song, G.; Mao, J.; Li, Y.; Ji, Z.; Chen, D.; Song, A. Internal Defect Detection of Overhead Aluminum Conductor Composite Core Transmission Lines With an Inspection Robot and Computer Vision. *IEEE Trans. Instrum. Meas.* **2023**, *72*, 1–16, doi:10.1109/TIM.2023.3265104.

86. Xuan, Y.; Zhang, X.-Y.; Li, C.; Wang, H.; Mu, C. LAM-YOLOv11 for UAV Transmission Line Inspection: Overcoming Environmental Challenges with Enhanced Detection Efficiency. *Multimed. Syst.* **2025**, *31*, 110, doi:10.1007/s00530-025-01702-y.

87. Gong, Y.; Gao, L.; Zhang, H.; Liu, H.; Zhu, Y.; Yang, Y. LARNet-SAP-YOLOv11: A Joint Model for Image Restoration and Corrosion Defect Detection of Transmission Line Fittings Under Multiple Adverse Weather Conditions. *IEEE Access* **2025**, *13*, 139310–139329, doi:10.1109/ACCESS.2025.3596300.

88. Choudhary, S.; Saurav, S.; Gidde, P.; Saini, R.; Singh, S. LPC-Det: Attention-Based Lightweight Object Detector for Power Line Component Detection in UAV Images. *Comput. Electr. Eng.* **2025**, *126*, 110476, doi:10.1016/j.compeleceng.2025.110476.

89. Yuan, J.; Hu, L.; Hu, Q. LRA-YOLO: A Lightweight Power Equipment Detection Algorithm Based on Large Receptive Field and Attention Guidance. *Information* **2025**, *16*, 736, doi:10.3390/info16090736.

90. Tran, D.K.; Nguyen, V.N.; Roverso, D.; Jenssen, R.; Kampffmeyer, M. LSNetv2: Improving Weakly Supervised Power Line Detection with Bipartite Matching. *Expert Syst. Appl.* **2024**, *250*, 123773, doi:10.1016/j.eswa.2024.123773.

91. Li, K.; Liu, M.; Wang, F.; Guo, X.; Han, G.; Bai, X.; Liu, C. Learning to Utilize Multi-Scale Feature Information for Crisp Power Line Detection. *Electronics* **2025**, *14*, 2175, doi:10.3390/electronics14112175.

92. Ji, H.; Chen, X.; Bai, J.; Gong, C. Lightweight SCL-YOLOv8: A High-Performance Model for Transmission Line Foreign Object Detection. *Sensors* **2025**, *25*, 5147, doi:10.3390/s25165147.

93. Pei, S.; Zhang, H.; Zhu, Y.; Hu, C. Lightweight Transmission Line Defect Identification Method Based on OFN Network and Distillation Method. *IET Image Process.* **2024**, *18*, 3518–3529, doi:10.1049/ipr2.13191.

94. Zhai, Y.; Hu, Z.; Wang, Q.; Yang, Q.; Yang, K. Multi-Geometric Reasoning Network for Insulator Defect Detection of Electric Transmission Lines. *Sensors* **2022**, *22*, 6102, doi:10.3390/s22166102.

95. Manninen, H.; Ramlal, C.J.; Singh, A.; Kilter, J.; Landsberg, M. Multi-Stage Deep Learning Networks for Automated Assessment of Electricity Transmission Infrastructure Using Fly-by Images. *Electr. Power Syst. Res.* **2022**, *209*, 107948, doi:10.1016/j.epsr.2022.107948.

96. Yang, Z.; Xu, X.; Wang, K.; Li, X.; Ma, C. Multitarget Detection of Transmission Lines Based on DANet and YOLOv4. *Sci. Program.* **2021**, *2021*, 1–12, doi:10.1155/2021/6235452.

97. Guo, Q.; Xiao, J.; Hu, X. New Keypoint Matching Method Using Local Convolutional Features for Power Transmission Line Icing Monitoring. *Sensors* **2018**, *18*, 698, doi:10.3390/s18030698.

98. Damodaran, S.; Shanmugam, L.; Swaroopan, N.M.J. Overhead Power Line Detection from Aerial Images Using Segmentation Approaches. *Automatika* **2024**, *65*, 261–288, doi:10.1080/00051144.2023.2296798.

99. Chen, J.; Zhang, X.; Feng, D.; Li, J.; Zhu, L. PLD-DETR: A Method for Defect Inspection of Power Transmission Lines. *Electronics* **2025**, *14*, 4107, doi:10.3390/electronics14204107.

100. Pang, F.; Jia, C.; Li, Y.; Lei, C.; Chen, X. Pi-Score: An Estimation Strategy of the Class Prior in Positive-Unlabeled Learning for Electrical Insulator Defect Detection with Incomplete Annotations. *J. Sensors* **2023**, *2023*, doi:10.1155/2023/6696721.

101. Yang, L.; Fan, J.; Huo, B.; Li, E.; Liu, Y. PLE-Net: Automatic Power Line Extraction Method Using Deep Learning from Aerial Images. *Expert Syst. Appl.* **2022**, *198*, 116771, doi:10.1016/j.eswa.2022.116771.

102. Jaffari, R.; Hashmani, M.A.; Reyes-Aldasoro, C.C.; Junejo, A.Z.; Taib, H.; Abdullah, M.N.B. PLPose: An Efficient Framework for Detecting Power Lines via Key Points-Based Pose Estimation. *J. King Saud Univ. - Comput. Inf. Sci.* **2023**, *35*, 101615, doi:10.1016/j.jksuci.2023.101615.

103. Yuan, X.; Xiao, Y.; Zhang, W.; Qiao, J.; Wei, G.; Zheng, Y. PLRefiner: Discrete Diffusion Model for Power Line Segmentation Refinement. *IEEE Sens. J.* **2025**, *25*, 38547–38558, doi:10.1109/JSEN.2025.3607978.

104. Yi, J.; Mao, J.; Zhang, H.; Zeng, K.; Tao, Z.; Zhong, H.; Wang, S.; Wang, Y. PSTL-Net: A Patchwise Self-Texture-Learning Network for Transmission Line Inspection. *IEEE Trans. Instrum. Meas.* **2024**, *73*, 1–14, doi:10.1109/TIM.2023.3341118.

105. Zhao, L.; Yao, H.; Fan, Y.; Ma, H.; Li, Z.; Tian, M. Power Line Detection for Aerial Images Using Object-Based Markov Random Field With Discrete Multineighborhood System. *IEEE Geosci. Remote Sens. Lett.* **2024**, *21*, 1–5, doi:10.1109/LGRS.2023.3345333.

106. Zhao, L.; Wang, X.; Yao, H.; Tian, M.; Jian, Z. Power Line Extraction From Aerial Images Using Object-Based Markov Random Field With Anisotropic Weighted Penalty. *IEEE Access* **2019**, *7*, 125333–125356, doi:10.1109/ACCESS.2019.2939025.

107. Xi, S.; Zhang, Z.; Niu, Y.; Li, H.; Zhang, Q. Power Line Extraction and Tree Risk Detection Based on Airborne LiDAR. *Sensors* **2023**, *23*, 8233, doi:10.3390/s23198233.

108. Wei, N.; Jin, Y.; Dan, Z.; Liu, N.; Peng, F.; Sun, S. Power Line Segmentation by Multilevel Attention From Hough Domain. *IEEE Trans. Instrum. Meas.* **2025**, *74*, 1–11, doi:10.1109/TIM.2024.3522395.

109. Xu, C.; Li, Q.; Zhou, Q.; Zhang, S.; Yu, D.; Ma, Y. Power Line-Guided Automatic Electric Transmission Line Inspection System. *IEEE Trans. Instrum. Meas.* **2022**, *71*, 1–18, doi:10.1109/TIM.2022.3169555.

110. Lu, G.; Li, B.; Chen, Y.; Qu, S.; Cheng, T.; Zhou, J. Precision in Aerial Surveillance: Integrating YOLOv8 With PConv and CoT for Accurate Insulator Defect Detection. *IEEE Access* **2025**, *13*, 49062–49075, doi:10.1109/ACCESS.2025.3551289.

111. Deng, Z.; Li, X.; Yang, R. RML-YOLO: An Insulator Defect Detection Method for UAV Aerial Images. *Appl. Sci.* **2025**, *15*, 6117, doi:10.3390/app15116117.

112. Shuang, F.; Han, S.; Li, Y.; Lu, T. RSIn-Dataset: An UAV-Based Insulator Detection Aerial Images Dataset and Benchmark. *Drones* **2023**, *7*, 125, doi:10.3390/drones7020125.

113. Ayoub, N.; Schneider-Kamp, P. Real-Time On-Board Deep Learning Fault Detection for Autonomous UAV Inspections. *Electronics* **2021**, *10*, 1091, doi:10.3390/electronics10091091.

114. Surantha, N. Real-Time Overhead Power Line Component Detection on Edge Computing Platforms. *Computers* **2025**, *14*, 134, doi:10.3390/computers14040134.

115. Zhang, J.; Guan, H.; Cai, Q.; Zhang, X.; Shao, H.; Qu, S. Research on Crimp Pitch Measurement for Overhead Transmission Line Based on Image Processing With Composite Algorithm. *Eng. Reports* **2025**, *7*, doi:10.1002/eng2.70124.

116. Dong, X.; Wan, Y.; Zhu, Y.; Ji, C. Research on Detection of Icing Cover Transmission Lines Under Different Weather Conditions Based on Wide-Field Dynamic Convolutional Network LDKA-NET. *Appl. Sci.* **2024**, *14*, 11486, doi:10.3390/app142411486.

117. Du, F.; Jiao, S.; Chu, K. Research on Safety Detection of Transmission Line Disaster Prevention Based on Improved Lightweight Convolutional Neural Network. *Machines* **2022**, *10*, 588, doi:10.3390/machines10070588.

118. Yang, L.; Yang, Q.; Zhong, Y.; Zhang, L.; Ren, X. Research on the Application Technology of Transmission Line Icing Segmentation Detection Model Based on Mamba's Visual Neural Network. *Discov. Appl. Sci.* **2025**, *7*, 1352, doi:10.1007/s42452-025-07877-3.

119. Wu, K.; Chen, Y.; Lu, Y.; Yang, Z.; Yuan, J.; Zheng, E. SOD-YOLO: A High-Precision Detection of Small Targets on High-Voltage Transmission Lines. *Electron.* **2024**, *13*, doi:10.3390/electronics13071371.

120. Lv, W.; Nie, M.; Yue, Y.; Liang, Y.; Wang, L.; Luo, Q.; Li, Q. STF-Net: A Model for Detecting Transmission Line Fittings in UAV Images. *Electr. Eng.* **2025**, *107*, 13119–13135, doi:10.1007/s00202-025-03198-1.

121. Lu, L.; Wang, L.; Wu, S.; Zu, S.; Ai, Y.; Song, B. Semantic Segmentation of Key Categories in Transmission Line Corridor Point Clouds Based on EMAFL-PTv3. *Electronics* **2025**, *14*, 650, doi:10.3390/electronics14040650.

122. Wang, L.; Chen, Z.; Hua, D.; Zheng, Z. Semantic Segmentation of Transmission Lines and Their Accessories Based on UAV-Taken Images. *IEEE Access* **2019**, *7*, 80829–80839, doi:10.1109/ACCESS.2019.2923024.

123. Hu, Z.; Zhai, B.; Zhao, Z.; Zhai, Y.; Wang, Q.; Yang, K. State-Space-Model-Guided Deep Feature Perception Network for Insulator Defect Detection in High-Resolution Aerial Images. *IEEE Trans. Geosci. Remote Sens.* **2025**, *63*, doi:10.1109/TGRS.2025.3584663.

124. Lu, Y.; Zheng, E.; Chen, Y.; Wu, K.; Yang, Z.; Yuan, J.; Xie, M. Stower-13: A Multi-View Inspection Image Dataset for the Automatic Classification and Naming of Tension Towers. *Electron.* **2024**, *13*, doi:10.3390/electronics13101858.

125. Ye, Y.; Tan, G.; Liu, Q.; Liu, L.; Chu, J.; Wen, B.; Li, L. TSSSKD-YOLO: An Intelligent Classification and Defect Detection Method of Insulators on Transmission Lines by Fusing Knowledge Distillation in Multiple Scenarios. *Multimed. Syst.* **2025**, *31*, 183, doi:10.1007/s00530-025-01772-y.

126. Xie, M.; He, Y.; Zhang, G.; Tang, C.; Ye, H.; Shen, J. TTower-345: A Multi-Categories Multi-Perspectives Benchmark for Automatic Naming of Transmission Line Inspection Photos. *Int. J. Adv. Mechatron. Syst.* **2024**, *11*, 11–25, doi:10.1504/IJAMECHS.2024.10063115.

127. Wang, Y.; Tian, H.; Yin, T.; Song, Z.; Hauwa, A.S.; Zhang, H.; Gao, S.; Zhou, L. The Transmission Line Foreign Body Detection Algorithm Based on Weighted Spatial Attention. *Front. Neurorobot.* **2024**, *18*, doi:10.3389/fnbot.2024.1424158.

128. Liu, C.; Liu, J.; Wu, Y.; Sun, Z. Thermal Defect Detection of Electrical Components in Transmission Lines Based on Infrared Images and Deep Learning†. *IEEJ Trans. Electr. Electron. Eng.* **2025**, *20*, 1730–1744, doi:10.1002/tee.70060.

129. Liu, X.; Miao, X.; Jiang, H.; Chen, J.; Wu, M.; Chen, Z. Tower Masking MIM: A Self-Supervised Pretraining Method for Power Line Inspection. *IEEE Trans. Ind. Informatics* **2024**, *20*, 513–523, doi:10.1109/TII.2023.3268479.

130. Yang, X.; Liu, K.; Guan, R.; Yu, J.; Liu, Y. Transformer-Based Detection of Hidden Hazards in Power Transmission Lines for UAV Inspection. *IEEE Access* **2025**, *13*, 150992–151000, doi:10.1109/ACCESS.2025.3603163.

131. Zhang, K.; Zhou, R.; Wang, J.; Xiao, Y.; Guo, X.; Shi, C. Transmission Line Component Defect Detection Based on UAV Patrol Images: A Self-Supervised HC-ViT Method. *IEEE Trans. Syst. Man, Cybern. Syst.* **2024**, *54*, 6510–6521, doi:10.1109/TSMC.2024.3386873.

132. Wang, Y.; Zhou, W.; Qian, X. Transmission Line Detection Through Auxiliary Feature Registration With Knowledge Distillation. *IEEE Trans. Autom. Sci. Eng.* **2025**, *22*, 9413–9425, doi:10.1109/TASE.2024.3506617.

133. Qiang, H.; Tao, Z.; Ye, B.; Yang, R.; Xu, W. Transmission Line Fault Detection and Classification Based on Improved YOLOv8s. *Electronics* **2023**, *12*, 4537, doi:10.3390/electronics12214537.

134. Zhao, L.; Liu, C.; Zhang, Z.; Qu, H. Transmission Line Object Detection Method Based on Label Adaptive Allocation. *Mathematics* **2022**, *10*, 2150, doi:10.3390/math10122150.

135. Han, G.; Wang, R.; Yuan, Q.; Zhao, L.; Li, S.; Zhang, M.; He, M.; Qin, L. Typical Fault Detection on Drone Images of Transmission Lines Based on Lightweight Structure and Feature-Balanced Network. *Drones* **2023**, *7*, 638, doi:10.3390/drones7100638.

136. Xu, C.; Wang, J.; Ding, Y.; Zheng, C. UAV Power Line Inspection Strategy Based on SAC Algorithm. *Electr. Power Syst. Res.* **2025**, *248*, 111925, doi:10.1016/j.epsr.2025.111925.

137. Liu, Y.; Qu, Z.; Zheng, G.; Ye, J. URMCNet: An Uncertainty Region Modeling and Calibration Network for Power Line Inspection. *IEEE Trans. Autom. Sci. Eng.* **2025**, *22*, 21242–21255, doi:10.1109/TASE.2025.3606206.

138. Li, J.; Chen, J.; Huang, M.; Li, L.; Tu, J.; Li, J.; Hu, T. Urban Power Line Extraction from Mobile LiDAR Point Clouds Based on Local Line-Plane Seperation Model and Global Refinement. *Meas. Sci. Technol.* **2025**, *36*, 36121, doi:10.1088/1361-6501/adb203.

139. Cano-Solis, M.; Ballesteros, J.R.; Branch-Bedoya, J.W. VEPL Dataset: A Vegetation Encroachment in Power Line Corridors Dataset for Semantic Segmentation of Drone Aerial Orthomosaics. *Data* **2023**, *8*, 128, doi:10.3390/data8080128.

140. Zhang, K.; Yang, J.; Wang, J.; Zheng, Z.; Sheng, X.; Zhang, N. VLF-DETR: Integrating Vision-Language and High-Frequency Features for Transmission Line Defect Detection. *IEEE Trans. Instrum. Meas.* **2025**, *74*, 1–15, doi:10.1109/TIM.2025.3586346.

141. Yang, L.; Fan, J.; Xu, S.; Li, E.; Liu, Y. Vision-Based Power Line Segmentation With an Attention Fusion Network. *IEEE Sens. J.* **2022**, *22*, 8196–8205, doi:10.1109/JSEN.2022.3157336.

142. Liu, K.; Zhong, L. Object Detection of UAV Power Line Inspection Images Based on Federated Learning. In Proceedings of the 2022 IEEE 5th International Electrical and Energy Conference (CIEEC); IEEE, May 2022; pp. 2372–2377.

143. Chen, J.; Shi, P.; Xu, M.; Xin, Y.; Fan, X.; Zhang, J. WCANet: An Efficient and Lightweight Weight Coordinated Adaptive Detection Network for UAV Inspection of Transmission Line Accessories. *Drones* **2025**, *9*, 318, doi:10.3390/drones9040318.

144. Kyem, B.A.; Asamoah, J.K.; Huang, Y.; Aboah, A. Weather-Adaptive Synthetic Data Generation for Enhanced Power Line Inspection Using StarGAN. *IEEE Access* **2024**, *12*, 193882–193901, doi:10.1109/ACCESS.2024.3520120.

145. Liu, Y.; Huang, X.; Liu, D. Weather-Domain Transfer-Based Attention YOLO for Multi-Domain Insulator Defect Detection and Classification in UAV Images. *Entropy* **2024**, *26*, 136, doi:10.3390/e26020136.

146. Farooq, U.; Yang, F.; Shahzadi, M.; Ali, U.; Li, Z. YOLOv8-IDX: Optimized Deep Learning Model for Transmission Line Insulator-Defect Detection. *Electronics* **2025**, *14*, 1828, doi:10.3390/electronics14091828.
147. Benelmostafa, B.-E.; Aitelhaj, R.; Medromi, H. YOLOv8-ERFD-AP: A Novel Domain Generalization Model for UAV-Based Insulator Inspection Under Adverse Weather Conditions. *IEEE Access* **2025**, *13*, 135336–135358, doi:10.1109/ACCESS.2025.3593201.
148. Jiao, R.; Liu, J.; Li, K.; Qiao, R.; Liu, Y.; Zhang, W. YOLO–DTAD: Dynamic Task Alignment Detection Model for Multicategory Power Defects Image. *IEEE Trans. Instrum. Meas.* **2025**, *74*, 1–14, doi:10.1109/TIM.2025.3541692.
